# Laterally π‑Extended Polyhelicenes

**DOI:** 10.1021/jacs.5c15494

**Published:** 2025-11-14

**Authors:** Hao Wu, Zijie Qiu, Guanzhao Wen, Antoine Hinaut, Koji Harano, Robert Graf, Deborah Prezzi, Lilian Estaque, Yu-Liang Tsai, Dieter Schollmeyer, Grégory Pieters, Elisa Molinari, Rémy Pawlak, Ernst Meyer, Koji Kimoto, Hai I. Wang, Mischa Bonn, Klaus Müllen, Akimitsu Narita

**Affiliations:** † Max Planck Institute for Polymer Research, Ackermannweg 10, Mainz 55128, Germany; ‡ School of Science and Engineering, Guangdong Basic Research Center of Excellence for Aggregate Science, Shenzhen Institute of Aggregate Science and Technology, 407605 The Chinese University of Hong Kong, Shenzhen (CUHK-Shenzhen), Shenzhen 518172, P. R. China; § Department of Physics, University of Basel, Klingelbergstrasse 82, Basel 4056, Switzerland; ∥ Center for Basic Research on Materials, 52747 National Institute for Materials Science, 1-1 Namiki, Tsukuba, Ibaraki 305-0044, Japan; ⊥ Research Center for Autonomous Systems Materialogy (ASMat), Institute of Integrated Research, Institute of Science Tokyo, Yokohama, Kanagawa 226-8501, Japan; # Istituto Nanoscienze, CNR, via G. Campi 213/a, Modena 41125, Italy; ∇ Université Paris-Saclay, CEA, INRAE, Département Médicaments et Technologies pour la Santé (DMTS), SCBM, Gif-sur-Yvette F-91191, France; ○ Department of Chemistry, Johannes Gutenberg University Mainz, Duesbergweg 10-14, Mainz 55128, Germany; ◆ Dipartimento di Scienze Fisiche, Informatiche e Matematiche, Università di Modena e Reggio Emilia, Modena 41125, Italy; ¶ Nanophotonics, Debye Institute for Nanomaterials Science, Utrecht University, Princetonplein 1, Utrecht 3584 CC, The Netherlands; †† Organic and Carbon Nanomaterials Unit, 508336 Okinawa Institute of Science and Technology Graduate University, 1919-1 Tancha, Onna-son, Kunigami-gun, Okinawa 904-0495, Japan

## Abstract

Helically coiled, semiconducting graphenic nanostructures show exceptional promise for nanoelectronics, yet their synthesis has remained challenging due to their inherently strained backbone and the difficulties associated with structural characterization. In this work, we demonstrate the synthesis and characterization of laterally π-extended polyhelicenes (EPHs), achieved through regioselective cyclodehydrogenation. Spectroscopic and microscopic analyses, including mass spectrometry, solid-state NMR, scanning-probe microscopy, and transmission electron microscopy, confirm the well-defined helical, layered architecture of the EPHs. Ultrafast terahertz spectroscopy reveals pronounced intrahelix photoconductivity, demonstrating their potential as carbon-based nanoscale conductors. The scalable synthetic approach described in this work unlocks the application potential of carbon-based helical nanostructures, paving the way for nanoinductors in nanoscale solenoids, spin-selective electronics, and future high-frequency nanoelectronic devices.

## Graphical abstract

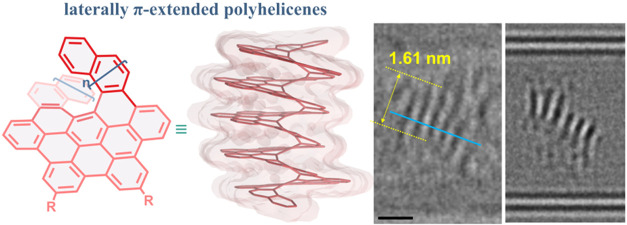

## Introduction

Helicenes are a fascinating family of organic molecules distinguished by their spiral-shaped structures, resembling molecular springs.^1–3^ Their structure is made up of a series of *ortho*-fused aromatic rings, and this helically extended π-conjugation not only imparts chirality but also unique (opto)­electronic properties, making helicenes valuable for applications ranging from molecular recognition^4^ and asymmetric catalysis^5,6^ to chiroptical devices^7,8^ and spin filters.^9,10^ Accordingly, the unique three-dimensional structure and intriguing properties of helicenes have prompted much work over many decades.^3^

However, conventional helicenes face two critical limitations: (1) their small molecular size limits the π-conjugation, restricting the optical properties and charge or spin transport capabilities, and (2) their extension to longer helical lengths, i.e., polyhelicenes, remains fundamentally inaccessible due to extreme backbone strain and lack of synthetic methods achieving the required regioselectivity.^11^ In fact, it took nearly 40 years until [16]­helicene, the highest carbohelicene currently known in the literature with 16 angularly fused benzene rings, was reported by Murase and Fujita in 2015,^12^ after [14]­helicene by Martin and Baes in 1975.^13^ Recent advances in helicenes with laterally extended π-conjugation have improved photoluminescence^14–18^ and chiroptical responses.^17,19–22^ Tanaka reported a π-extended [13]­helicene featuring three polycyclic layers.^23^ Nevertheless, these systems remain discrete molecules incapable of supporting long-range charge transport required for nanoelectronic applications, leaving a huge gap between such “small” molecules and their polymer homologues, namely laterally π-extended polyhelicenes (EPHs). In addition to the synthetic challenge, the structural characterization of EPHs faces severe difficulties due to their complex 3D macromolecular structures with vanishing solubility.

In this work, we report the first synthesis and structure proof of EPHs. Our approach is based on the oxidative cyclodehydrogenation of naphthylene-bridged precursors. The π-extended [12]­helicene-based model dimer E[12]H **2** and EPH **4** were synthesized from precursors **1** and **3**, respectively (see Figure 1). Virtually perfect regioselectivity was achieved by involving the reactive α-positions of the naphthalene units^20,24,25^ and thus overcoming the steric hindrance toward the strained structures of E[12]H **2** and EPH **4**. Structural proof of EPH **4** was obtained by the matrix-assisted laser desorption/ionization time-of-flight (MALDI-TOF) mass spectrometry (MS) and solid-state NMR spectroscopy. Moreover, the helical layered structure of EPH **4** was revealed by low-temperature scanning probe microscopy (LT-SPM) and high-resolution transmission electron microscopy (HR-TEM). Photoconductivity studies by ultrafast optical pump–terahertz probe (OPTP) spectroscopy elucidated an outstanding intrahelix conductivity of EPH **4**, which was absent for E[12]H **2**. EPHs possess helically coiled graphenic nanostructures, which have been proposed as next-generation nanosolenoid inductors.^26^ Therefore, the new EPHs pave the way toward carbon-based helical nanoconductors and inductors for future nanoelectronics.

**Figure 1:**
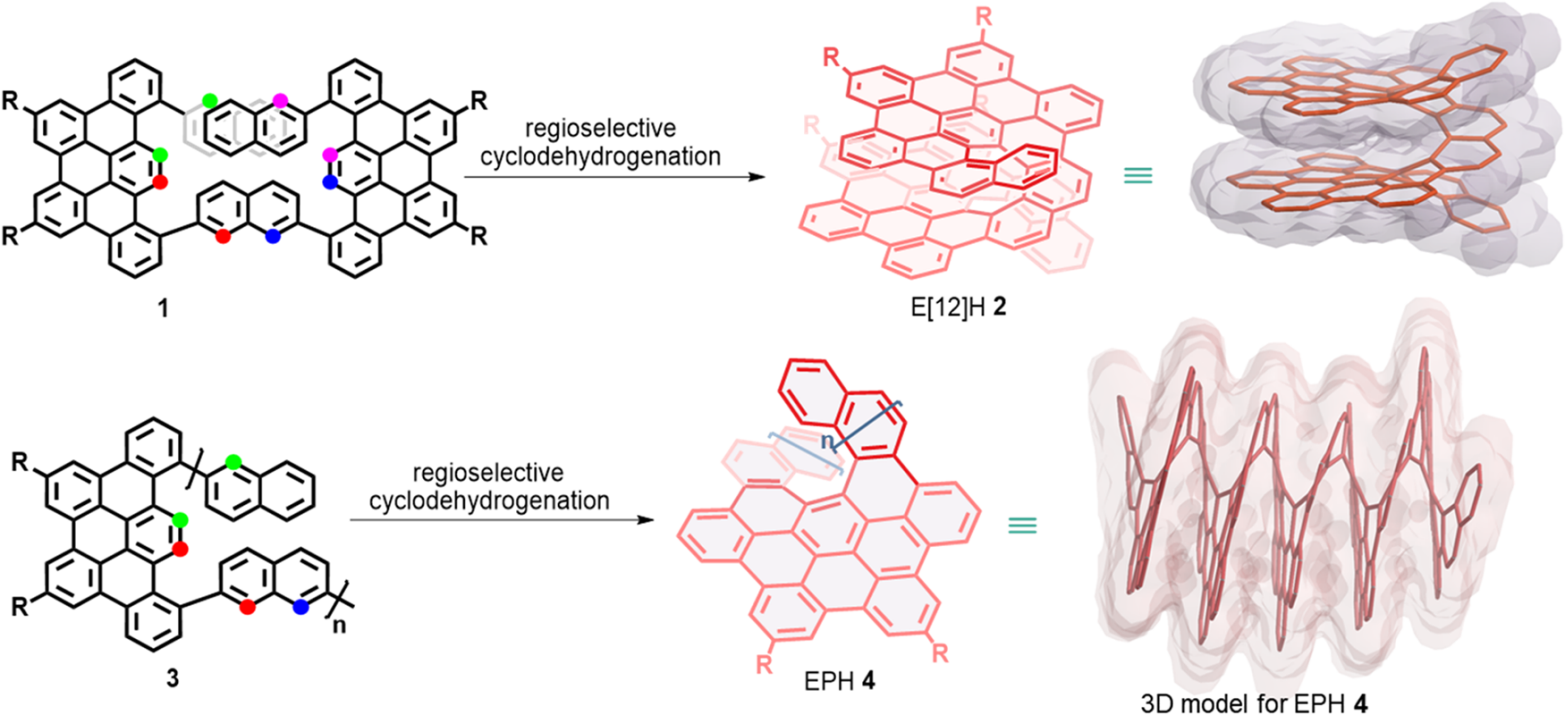
Synthesis of E[12]H **2** and EPH **4** by the regioselective cyclodehydrogenation reaction and their nonoptimized 3D models (when *n* = 5 for EPH **4**), showing the helically coiled structures and Connolly surfaces. R groups are omitted for clarity. The colored dots indicate the positions of C–C bond formation during regioselective cyclodehydrogenation.

## Results and Discussion

We synthesized E[12]H **2** as a model compound for EPH **4** by dehydrogenating precursor **1** consisting of two naphthyl-substituted tribenzo­[*fg*,*ij*,*rst*]­pentaphene (TBP) units,^25^ bridged by a naphthylene linker, initially using *t*Bu as solubilizing groups. Suzuki coupling of TBP dibromide **9a** with 1 equiv of 2-naphthylboronic acid provided mononaphthyl-substituted TBP **10a**, which was further coupled with naphthalene-2,7-diyldiboronic acid pinacol ester to yield precursor **1a** (Figure 2A). The oxidative cyclodehydrogenation of **1a** using 2,3-dichloro-5,6-dicyano-1,4-benzoquinone (DDQ) and triflic acid (TfOH) afforded a trace amount of E[12]H **2a**, the structure of which could be proven by the single-crystal X-ray analysis (CCDC 2396479, Figure S17). To improve the solubility of E[12]H **2**, we next used bulkier 2,4,4-trimethylpentan-2-yl groups as substituents. Suzuki coupling of 2,3-dibromo-1,4-bis­(trimethylsilyl)­benzene (**5b**) and 2,4,4-trimethylpentan-2-ylphenylboronic acid gave *ortho*-terphenyl **6b**. Subsequently, iodination of **6b** efficiently produced diiodide **7b**, which was then coupled with (2-bromophenyl)­boronic acid to obtain tetraphenylbenzene **8b**. The cyclodehydrogenation of **8b** using DDQ and TfOH yielded TBP dibromide **9b**, which exhibited far better solubility compared to **9a** in common organic solvents. Following a similar synthetic protocol as described above for **1a**, precursor **1b** was obtained in 9% yield over two steps from **9b**. Notably, the cyclodehydrogenation of **1b** yielded E[12]H **2b** as a sole detectable product with a very high isolated yield of 89% after purification by silica gel column chromatography. MALDI-TOF MS analysis of E[12]H **2b** displayed *m*/*z* = 1568.8133 (calculated exact mass of C_122_H_104_: 1568.8138) and an isotopic distribution pattern that is fully consistent with the simulated one. Well-resolved ^1^H and ^13^C NMR spectra could also be recorded for E[12]H **2b**, supporting its chemical structure (Figures S27–28).

**Figure 2:**
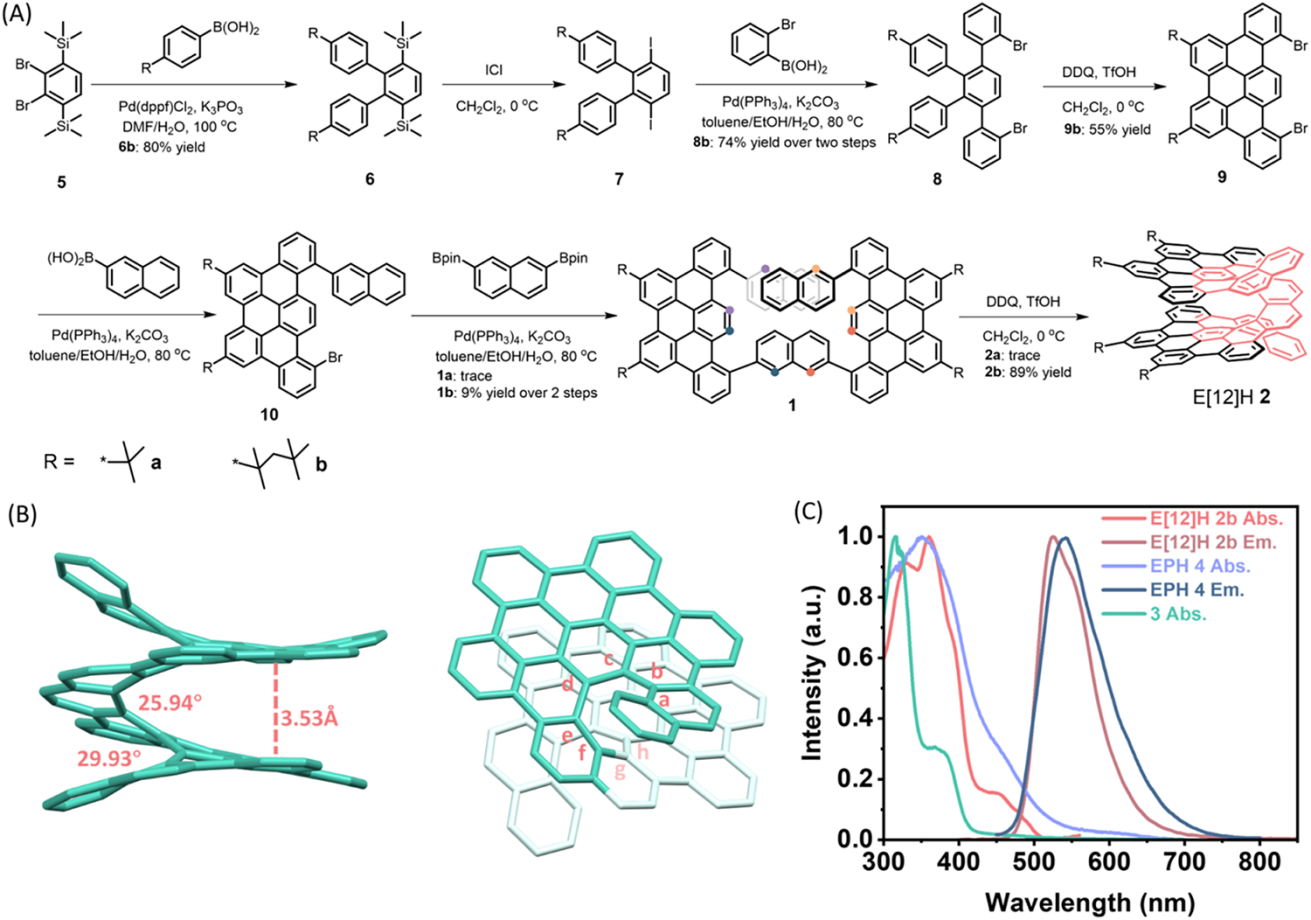
(A) Synthetic route to E[12]H **2**. The colored dots represent the positions of chemical bond formation from **1** to **2**. (B) Side-view and top-view of the single-crystal structure of E[12]H **2b**. All hydrogen atoms and the alkyl chains are omitted for clarity. (C) UV–vis absorption spectra of E[12]H **2b**, EPH **4**, and **3**, as well as emission spectra of E[12]H **2b** and EPH **4** in CH_2_Cl_2_.

A single crystal of E[12]H **2b** (CCDC 2396480) was grown by the slow diffusion of methanol vapor into its solution in dichloromethane. The three-layered [12]­helicene-based structure of E[12]H **2b** was confirmed by X-ray crystallography, verifying the remarkable regioselectivity in the cyclodehydrogenation of precursor **1b** (Figures 2B and S18). E[12]H **2b** possesses two almost parallel nanographene layers, showing a nearly AA stacking with the π–π distance of 3.53 Å, measured as the centroid-centroid distance between the TBP substructures. This indicates strong intramolecular π–π interactions, as also supported by the intramolecular noncovalent interaction (NCI) analyses (see Figure S16), potentially offering an intramolecular through-space charge transport channel.^27^ The torsional angle in middle (atoms d-e-f-g) segment in E[12]H **2b** was estimated to be 25.9°, which is smaller than that in our previous π-extended [7]­helicene (28.2°),^20^ pointing toward a more compressed helical pitch in E[12]H **2b**.

The UV–vis absorption spectra of E[12]H **2b** in dichloromethane (Figure 2C) displayed the absorption maximum at 360 nm and a lower-intensity band centered at 475 nm. Fluorescence measurements showed an emission with a maximum at 525 nm and an absolute fluorescence quantum yield of 0.14 in dichloromethane. This value is considerably higher than that of 0.006 reported for pristine [12]­helicene,^28^ which could be theoretically rationalized by the larger oscillator strengths of E[12]H **2b** for both absorption (S_0_ → S_1_) and fluorescence (S_1_ → S_0_) (Figures S12 and S13). Additionally, the resolution of E[12]H **2b** into its *P*/*M* enantiomers, with the calculated barrier of 87.8 kcal/mol (Figure S14), was achieved by chiral high-performance liquid chromatography (HPLC) (Figure S1). Chiroptical analyses by circular dichroism (CD) and circularly polarized luminescence (CPL) spectroscopies revealed mirror images between the two enantiomers and moderate absorption and luminescent dissymmetry factors of up to 0.001 (*g*_abs_) and 0.004 (*g*_lum_), respectively (Figure S3). These values are 1 order of magnitude smaller than those reported for π-extended [11]- and [13]­helicenes by Tanaka,^23^ but agreed well with the simulation by the density functional theory (DFT) calculations (Figure S15). Distinct arrangements of the electric and magnetic transition dipole moments are elucidated for the ground and excited states, which account for the higher *g*_lum_ value of E[12]H **2b** compared to *g*_abs_. The angles between the electric and magnetic transition dipole moments are revealed to be the major limiting factor, providing an insight into the future designs of π-extended helicenes with higher dissymmetry factors.

The improved solubility of TBP dibromide **9b** with the branched 2,4,4-trimethylpentan-2-yl chains enabled the preparation of polymer **3** through the A_2_B_2_-type Suzuki polymerization with naphthalene bisboronic ester **11** (Figure 3A). Among different conditions, the Pd­(OAc)_2_/Xphos catalytic system gave **3** in 65% yield, with the highest weight-average molecular weight (*M*_w_ = 8800, based on the size-exclusion chromatography analysis with the polystyrene standard, Figure S4). Subsequently, the oxidative cyclodehydrogenation of **3**, under the same conditions as the preparation of E[12]H **2b**, afforded EPH **4** in 82% yield. MALDI-TOF MS analysis of EPH **4** exhibited periodic signals corresponding to its oligomers ranging from trimer to heptamer with the backbones of [17]- to [37]­helicenes (Figure 3B). As expected for the A_2_B_2_-type Suzuki polymerization, oligomer species with remaining pinacol borate (Bpin) groups ([M+Bpin]^+^ and [M+2Bpin]^+^), as well as those lacking the terminal naphthy (Nap) groups [M–Nap]^+^ were also detected. Nonetheless, the former could be attributed to the detection of traces due to the higher ionization tendency, as confirmed by the absence of the quaternary carbon signal from Bpin in the solid-state NMR spectrum (*vide infra*). The isotopic distributions observed for different oligomer structures were in very good agreement with the simulated patterns (Figures 3C and S5), although some dehydrogenation apparently occurred during the measurements, requiring high laser intensity.

**Figure 3:**
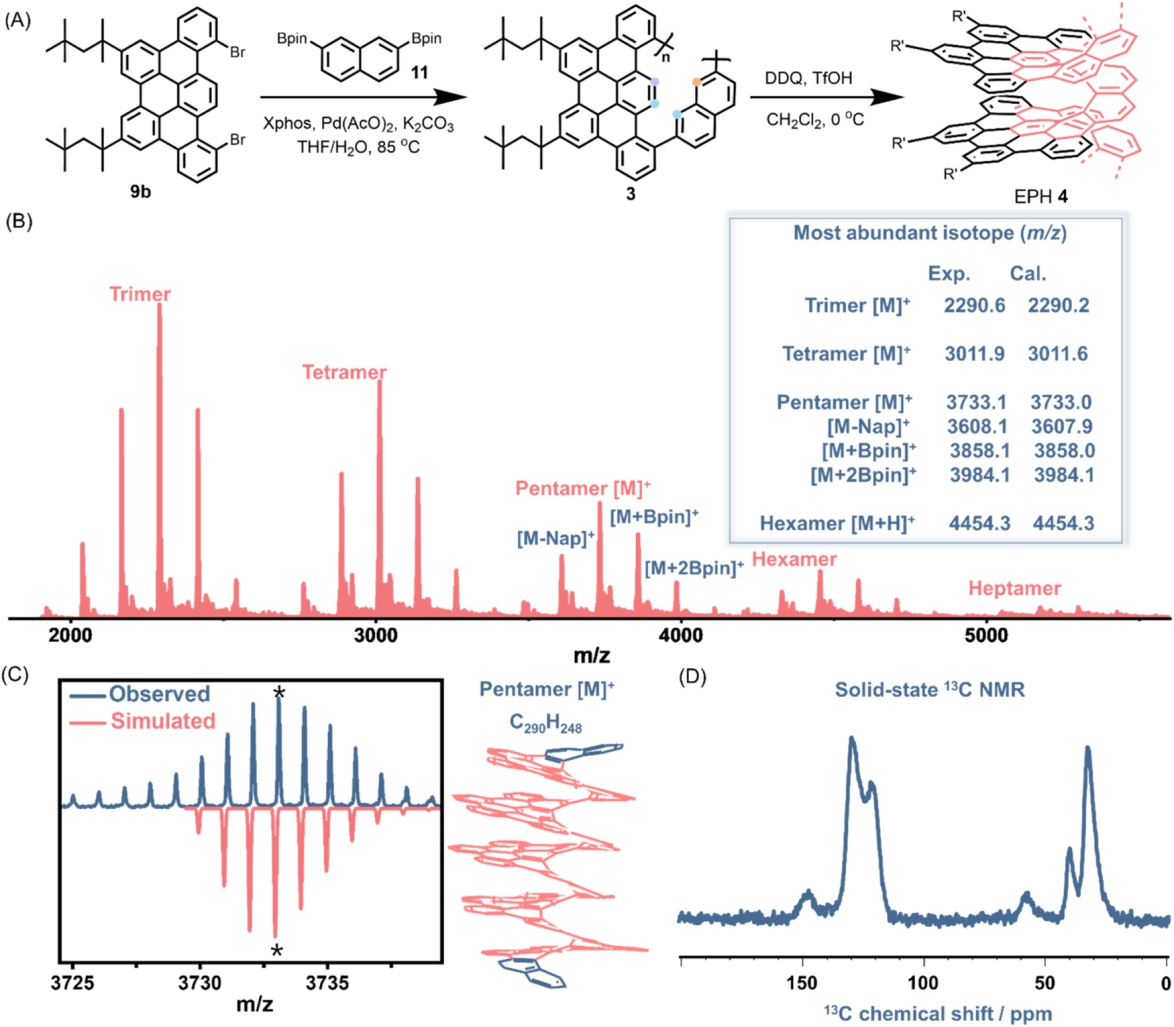
(A) Synthetic route to EPH **4**. The colored dots represent the positions of chemical bond formation from **3** to **4**. (B) MALDI-TOF mass spectra of EPH **4**. A list of observed and calculated mass values of the most abundant isotopes of the oligomers (as marked with * for the pentamer) is displayed on the right. (C) The experimental and simulated isotopic distribution of pentamer [M]^+^ along with its chemical structure. (D) Solid-state ^13^C CP-MAS NMR spectrum of EPH **4**.

The ^1^H Cross-Polarization-Magic Angle Spinning (CP-MAS) NMR spectrum of EPH **4** showed only two broad signals of aromatic and aliphatic protons (Figure S7), similar to the spectra of solution-synthesized graphene nanoribbons (GNRs)^29^ described in the literature, including those with helically coiled structures.^30^ Nonetheless, relatively more resolved signals were observed in the ^13^C CP-MAS NMR spectrum of EPH **4**, showing signals from the methyl, quaternary, and methylene carbons of the 2,4,4-trimethyl-pentane-2-yl chains at 32, 40, and 58 ppm, respectively, in the aliphatic region (Figure 3D). In the aromatic region, three peaks were identified at 122, 130, and 148 ppm, consistent with the ^13^C NMR spectrum of E[12]H **2b** (Figure S28). Moreover, there was no detectable signal between ∼60 and 110 ppm and >160 ppm, implying the absence of Bpin or unexpected oxygen-containing functional groups on the peripheral positions in the vicinity of aromatic protons and corroborating the structure of EPH **4**. Additional evidence for the successful cyclodehydrogenation of polymer **3** to EPH **4** was obtained by UV–vis absorption, emission, and FT-IR spectroscopic analyses (Figures 2C and S8), in line with previously reported GNRs.^30–35^ The UV–vis spectrum of EPH **4** were significantly red-shifted from that of polymer **3**, and exhibited a broad shoulders extending to ∼650 nm, compared to those of E[12]H **2b** up to ∼500 nm. The emission spectrum of EPH **4** is similar to, but broader than that of E[12]H **2b**, which might be due to predominant fluorescence of shorter oligomers and red-shifted emission from longer oligomers contained in EPH **4**, but this needs to be addressed by preparing EPH **4** with varied lengths and narrow molecular weight distributions.

To confirm the helical structure of EPH **4**, low-temperature (4.8 K) high-resolution SPM measurements were carried out on an Au(111) surface after their high-vacuum electrospray deposition (HV-ESD) (Figures 4 and S9–S10).^33,36–38^ After annealing the substrate at 100 °C in UHV to remove remaining solvents and other contaminants,^39^ linear structures with lengths of up to 30 nm were observed by scanning tunneling microscopy (STM), aligned along step edges of the Au(111) surface (Figures 4A and S10). The zoom-in STM (Figure 4B) and an atomic force microscopy (AFM) images obtained with the carbon monoxide terminated tip (CO-AFM)^40^ (Figure 4C) further show that these polymers have a periodic modulation, which we ascribe to apexes of the TBP substructures or the peripheral alkyl groups of EPH **4** (i.e., its helical pitch). The distance between these protrusions is approximately 0.40 nm, as indicated in the STM and CO-AFM profiles (Figure 4D), which is in very good agreement with the intramolecular π-π distance of 3.53 Å measured in the single crystal structure of E[12]H **2b** (Figure 2B), strongly supporting the formation of EPH **4**.

**Figure 4:**
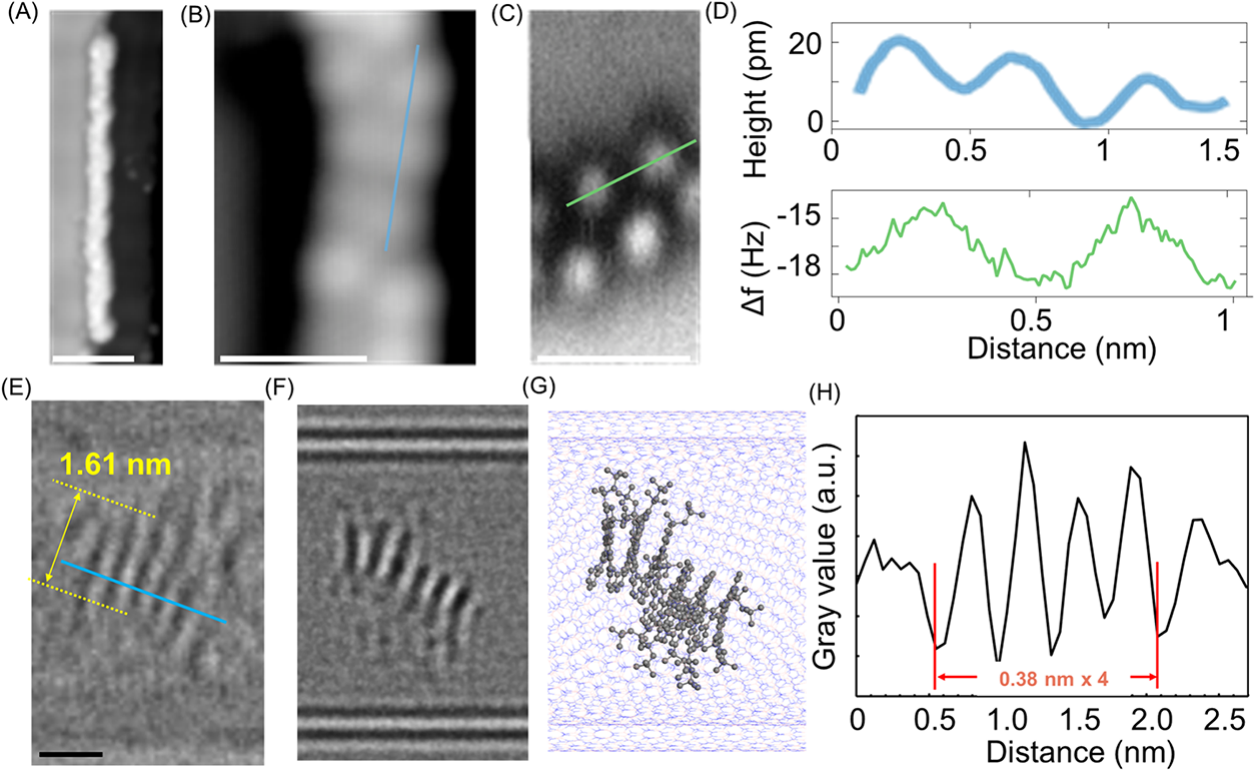
(A) STM topography of an isolated EPH **4** after HV-ESD on Au(111) followed by annealing at 100 °C (*I* = 1 pA, *U* = −400 mV). Scale bar: 5 nm. (B) Zoomed STM (*I* = 1 pA, *U* = −200 mV) and (C) CO-AFM images (*A* = 40 pm, *f* = 26 kHz) of EPH **4**. Scale bar: 1 nm. (D) Height and frequency shift profiles extracted from panels (B, C), respectively. (E) HR-TEM image of EPH **4** in a double-walled BNNT. Scale bar: 1 nm. (F) A TEM simulation image of heptameric EPH **4**. (G) An atomic-number-correlated molecular model corresponding to (F). (H) Intensity profile extracted from panel (E) (cyan line).

Furthermore, through the insertion of EPH **4** into boron nitride nanotubes (BNNTs), we achieved the visualization of an EPH by the high-resolution transmission electron microscope (HR-TEM) as a single-molecule image.^41,42^ By mixing open-ended BNNTs with EPH **4** in methanol/toluene, we successfully encapsulated EPH **4** inside the BNNTs with an inner diameter larger than 3 nm. In the TEM image of a debundled double-walled EPH@BNNT recorded at an accelerating voltage of 80 kV (Figures 4E and S11), an EPH molecule was observed to possess a width of 1.61 nm that matched the simulated TEM image (Figure 4F) derived from a theoretical model (Figure 4G), as well as the observed width of STM images (1.6–1.7 nm) (Figure S10A). Remarkably, periodic stripe-like contrasts along the tube axis were clearly observed, attributed to the overlap of carbon atoms in the π-conjugated structure of EPH viewed from the side, as seen in the simulated TEM image.^43^ Line profile analysis revealed that the distance between each layer was approximately 0.40 nm (Figure 4H), consistent with results obtained from STM and CO-AFM. Overall, these microscopic characterization results collectively indicated that EPH **4** with a helically coiled structure was successfully obtained.

The band structure of simplified EPH **4** calculated by the DFT shows a non-negligible dispersion along the helix and a reduced energy gap with respect to E[12]H **2** (from about 1.9 to 1.5 eV) (Figure S16), in line with optical measurements in which the red-shift of the absorption onset is observed. The plot of the frontier orbitals and the noncovalent interaction highlights that the π-conjugation along the helix is accompanied by π–π stacking interactions between the TBP substructures, which can collectively contribute to the band gap reduction.

To investigate the charge-carrier transport properties of EPH **4**, we performed photoconductivity studies using ultrafast optical pump terahertz probe (OPTP) spectroscopy. Figure 5A presents the transient photoconductivity of EPH **4** compared to those of TBP **9b** and E[12]H **2b**, all measured in thin films. While TBP **9b** and E[12]H **2b** show negligible photoconductivity due to their inability to support current, marking their molecular nature, EPH **4** demonstrates pronounced terahertz (THz) photoconductivity, indicating significant charge carrier generation and transport properties. Following the transient injection of free charge carriers, a fast ∼ps conductivity decay can be assigned to exciton formation, based on previous reports.^44,45^ This assignment is in line with imaginary conductivity-dominated dynamics from 5 ps onward. We note that EPH **4** dispersed in toluene exhibited no measurable photoconductivity, indicating that inter-EPH charge separation is essential for free carrier generation in the EPH films.^46,47^

**Figure 5:**
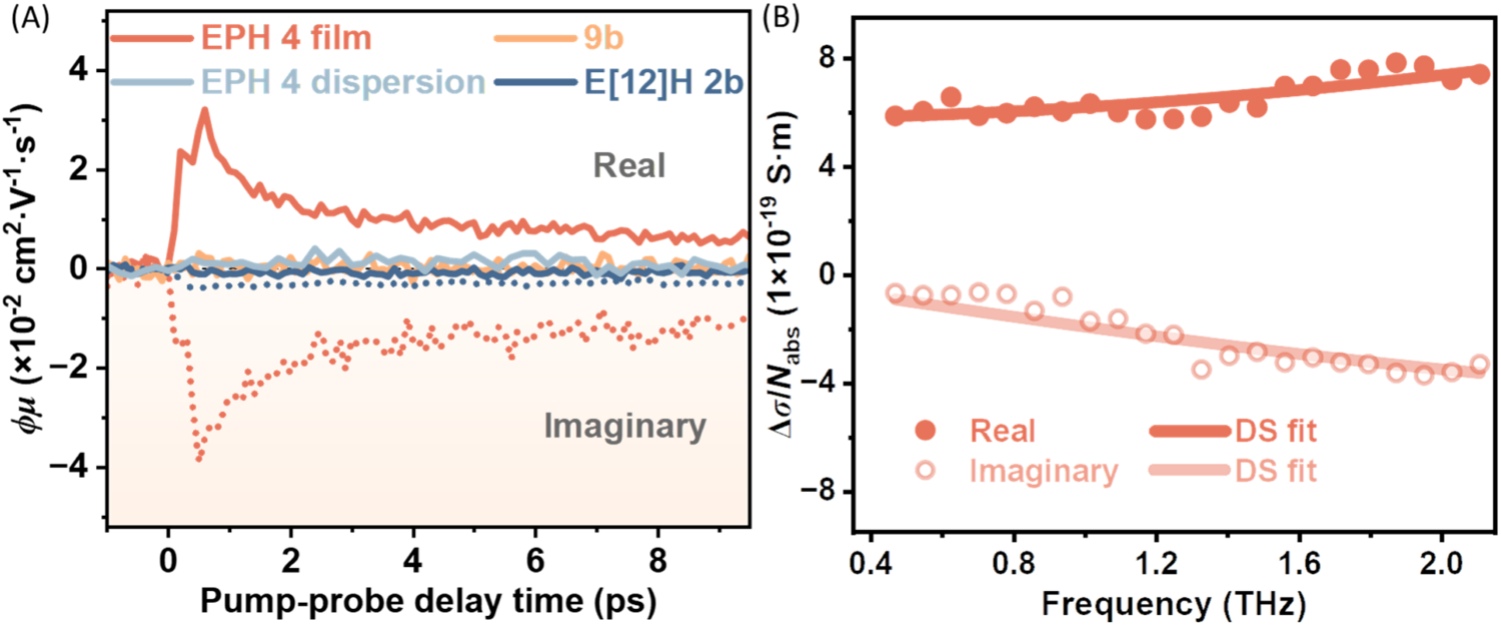
(A) Time-resolved complex terahertz photoconductivity of E[12]H **2b**, **9b**, and EPH **4** (film and dispersion in toluene) normalized to the absorbed photon density, following optical excitations by 400 nm laser pulses. (B) Frequency-resolved terahertz conductivity measured at ∼1.2 ps after photoexcitation for EPH **4**. The solid lines are fitted to the Drude–Smith model.

We further performed time-domain THz spectroscopic analysis at ∼1.2 ps following the free carrier injection. As presented in Figure 5B, the inferred frequency-resolved photoconductivity shows real-value-dominated photoconductivity, which unveils the free carrier-dominated dynamics at early times. Fitting the complex conductivity by the Drude-Smith model^48,49^ yields the charge scattering time τ = 16 ± 2 fs, and the parameter *c* = −0.81 ± 0.01, indicating that the charge transport is most likely via through-bond conduction along the π-conjugated backbone. The *c* parameter characterizes the probability of backscattering effects of charge carriers in EPHs due to their limited length or local structural deformation.^44^ By knowing these local transport parameters and approximating the charge carrier effective mass through the DFT calculations (m* = 0.4 ± 0.1 m_0_, see SI for more detail), a high short-range intrinsic (μ) and dc-limit carrier mobilities (μ_dc_) of 70 ± 20 and 13 ± 4 cm^2^ V^–1^ s^–1^ were estimated, following μ = *e*τ/m* and μ_dc_ = μ­(1 + *c*), respectively. The short-range mobility of EPH **4** is comparable to that of multiple solution-synthesized graphene nanoribbons (GNRs) in the literature (see Table S3), such as methoxy-substituted GNR (GNR-OMe; ∼60 cm^2^ V^–1^ s^–1^)^50^ and fjord-edge GNR (FGNR; ∼102 cm^2^ V^–1^ s^–1^).^51^ However, EPH **4** exhibits a higher mobility at the dc-limit, as a result of its relatively weaker backscattering effects (with *c* = −0.8), compared to other GNRs, which often have a *c* parameter close to −1. Considering the inferred excellent charge carrier mobility as well as the sufficiently high π-conjugation alone the EPH backbone, the charge transport can presumably occur via through-bond conduction, rather than through-space hopping.

## Conclusion

In summary, we achieved the synthesis of laterally π-extended polyhelicene (EPH), clearly elucidating its helical layered structure by high-resolution SPM and TEM. Our regioselective cyclodehydrogenation strategy has proved to be highly efficient for the formation of the polyhelicene backbone, holding promise for creating a broad range of related structures. The obtained EPH demonstrated a remarkable intrahelix conductivity, validating its potential as a nanoconductor. This represents the crucial first step toward developing carbon-based nanoinductors for nanoscale solenoid applications, which is essential for continued miniaturization of integrated circuits. The next step to this end will be the fabrication of EPH-based electrical devices and the characterization of their properties as nanoinductors by incorporating magnetic fields into the experiments. Furthermore, synthesis of enantiopure EPH is currently ongoing in our laboratories toward the development of carbon-based spin filters.

## Supplementary Material



## Data Availability

All data are available in the main text or the Supporting Information.

## Notes

Experimental details, characterization spectra of all synthesized compounds, single-crystal data, photophysical measurements, and computational details (PDF)
