# Supplementary material for: Laterally π‑Extended Polyhelicenes
Source: J Am Chem Soc. 2025 Nov 14;147(47):43842–9. doi: 10.1021/jacs.5c15494 (PMC12673593; doi:10.1021/jacs.5c15494)
Supplement: Supplementary file 1 [file ja5c15494_si_001.pdf]

Laterally π-Extended Polyhelicenes

Hao Wu,[1] Zijie Qiu,[2*] Guanzhao Wen,[1] Antoine Hinaut,[3] Koji Harano,[4,5] Robert Graf,[1] Deborah Prezzi,[6] Lilian Estaque,[7] Yu-Liang Tsai,[1] Dieter Schollmeyer,[8] Grégory Pieters,[7] Elisa Molinari,[6,9] Rémy Pawlak,[3] Ernst Meyer,[3] Koji Kimoto,[4] Hai I. Wang,[1,10] Mischa Bonn,[1] Klaus Müllen,[1,8*] Akimitsu Narita[1,11*]

[1] Max Planck Institute for Polymer Research, Ackermannweg 10, Mainz, 55128, Germany

[2] School of Science and Engineering, Guangdong Basic Research Center of Excellence for Aggregate Science, Shenzhen Institute of Aggregate Science and Technology, The Chinese University of Hong Kong, Shenzhen (CUHK-Shenzhen), Shenzhen, 518172, P.R. China

[3] Department of Physics, University of Basel, Klingelbergstrasse 82, Basel, 4056, Switzerland

[4] Center for Basic Research on Materials, National Institute for Materials Science, 1-1 Namiki, Tsukuba, Ibaraki, 305-0044, Japan

[5] Research Center for Autonomous Systems Materialogy (ASMat), Institute of Integrated Research, Institute of Science Tokyo, Yokohama, Kanagawa, 226–8501, Japan

[6] Istituto Nanoscienze, CNR, via G. Campi 213/a, Modena, 41125, Italy

[7] Université Paris-Saclay, CEA, INRAE, Département Médicaments et Technologies pour la Santé (DMTS), SCBM, Gif-sur-Yvette, F-91191, France

[8] Department of Chemistry, Johannes Gutenberg University Mainz, Duesbergweg 10-14, Mainz, 55128, Germany

[9] Dipartimento di Scienze Fisiche, Informatiche e Matematiche, Università di Modena e Reggio Emilia, Modena, 41125, Italy

[10] Nanophotonics, Debye Institute for Nanomaterials Science, Utrecht University, Princetonplein 1, Utrecht, 3584 CC, The Netherlands

[11] Organic and Carbon Nanomaterials Unit, Okinawa Institute of Science and Technology Graduate University, 1919-1 Tancha, Onna-son, Kunigami-gun, Okinawa, 904-0495, Japan

1. Experimental section

General Methods

All reactions working with air- or moisture-sensitive compounds were carried out under a nitrogen atmosphere using standard Schlenk line techniques. Unless otherwise noted, all starting materials were purchased from commercial sources and used without further purification. All other reagents were used as received. Preparative column chromatography was performed on silica gel from Merck with a grain size of 0.063-0.200 mm. Nuclear Magnetic Resonance (NMR) spectra were recorded on AVANCE 400 Bruker spectrometers. Abbreviations: s = singlet, d = doublet, t = triplet, q = quartet, m = multiplet, dd = doublet of doublets, br = broad signal. High-resolution mass spectrometry (HRMS) was performed on a SYNAPT G2 Si high-resolution time-of-flight mass (TOF) spectrometer (Waters Corp., Manchester, UK) by matrix-assisted laser desorption/ionization (MALDI). Analytical size exclusion chromatography (SEC) was performed on SDV PSS GPC columns using tetrahydrofuran (THF) as eluent at a temperature of 303 K. UV-Vis absorption spectra were recorded on a Perkin-Elmer Lambda 900 spectrophotometer using a 10 mm quartz cell. Fluorescence spectra were measured using an Edinburgh FS-5 spectrofluorimeter (with a SC-20 module), and a right-angle configuration was used. PLQY was determined using an integrating sphere (Edinburgh SC-30) on the Edinburgh FS-5 spectrofluorometer at 10^{-5} M in dichloromethane (DCM) solution. Chiral high-performance liquid chromatography (HPLC) was performed with a Daicel Chiralpak IE column. The circular dichroism (CD) spectra were collected on the JASCO J-1500 circular dichroism spectrometer at 298 K. The circularly polarized luminescence (CPL) measurements were performed using a JASCO CPL-300 at room temperature with 10 × 10 mm cells. Data pitch was set at 1 nm, scanning speed was set at 100 nm/min, and spectra displayed were mean values of 10 accumulations. Fourier transform infrared spectroscopy (FT-IR) measurement was conducted with a Bruker Tensor II FTIR spectrometer. X-ray crystallography was executed through Stoe IPDS 2T with Cu and Mo-X ray tubes and Oxford Cryostream.

Synthesis

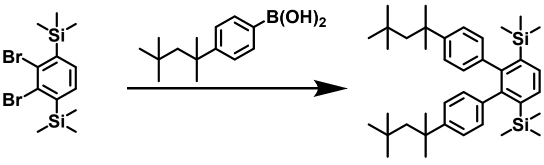

5 6b

[4,4''-bis(2,4,4-trimethylpentan-2-yl)-(1,1':2',1''-terphenyl)-3',6'-diyl]bis(trimethylsilane) (6b): Under an argon atmosphere, (2,3-dibromo-1,4-phenylene)bis(trimethylsilane) (**5**)[1] (5.80 g, 15.3 mmol), 4,4,5,5-tetramethyl-2-[4-(2,4,4-trimethylpentan-2-yl)phenyl]-1,3,2-dioxaborolane[2] (10.7 g, 33.5 mmol), $K_3PO_4 \cdot 7H_2O$ (16.0 g, 105 mmol) and [1,1'-bis(diphenylphosphino)ferrocene]dichloropalladium(II) (Pd(dppf)Cl_2), (1.11 g, 1.52 mmol) were dissolved in a mixture of dimethylformamide (DMF, 100 mL) and water (15 mL). The solution was degassed by bubbling with argon for 20 min, and then heated to 90 °C. After stirring overnight, the black mixture was cooled to room temperature and then water was added. The aqueous phase was extracted with hexane. The organic phases were washed with brine and water, and dried over $MgSO_4$. After removing the solvent by rotary evaporation, the residue was purified by silica gel column chromatography (hexane as eluent), affording **6b** as a white solid (7.32 g, 80% yield) .^{1}H NMR (400 MHz, CD_2Cl_2) δ 7.65 (s, 2H), 7.13 (d, J = 8.4 Hz, 4H), 6.88 (d, J = 8.3 Hz, 4H), 1.77 (s, 4H), 1.30 (s, 12H), 0.78 (s, 18H), 0.00 (s, 18H). ^{13}C NMR (101 MHz, CD_2Cl_2) δ 148.83, 148.17, 140.69, 139.75, 133.12, 131.15, 124.96, 56.83, 38.76, 32.85, 32.50, 32.20. HRMS (MALDI-TOF, positive) *m/z*: Calcd. For $C_{40}H_{62}Si_2$: 598.4390; Found: 598.4385 $[M]^+$.

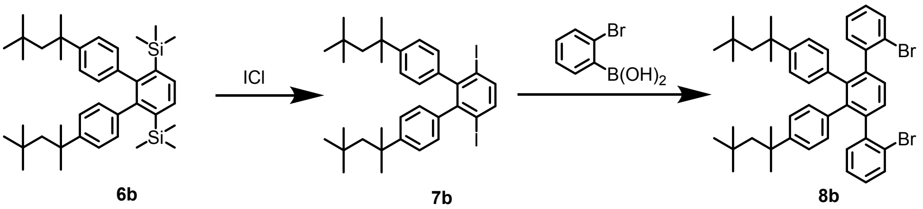

6b 7b 8b

2-bromo-4'-(2-bromophenyl)-4''-(2,4,4-trimethylpentan-2-yl)-3'-[4-(2,4,4-trimethylpentan-2-yl)phenyl]-1,1':2',1''-terphenyl (8b): Compound **6b** (6.53 g, 11.4 mmol) was dissolved in 80 mL of dry dichloromethane (DCM), and the solution was degassed by argon bubbling for 20 min. Afterwards, the solution was sealed under argon and cooled to 0 °C. A DCM solution of iodine monochloride (ICl) (7.42 g, 45.8

mmol) was added dropwise. After stirring at 0 °C for 1 h, the reaction was quenched by the addition of an aqueous solution of Na_2SO_3. The aqueous phase was extracted with DCM. The combined organic phases were washed with brine and water, and dried over $MgSO_4$. After the solvents were removed by rotary evaporation, the residue was purified by silica gel column chromatography (hexane:DCM = 10:1 as eluent), affording the crude compound **7b** as a white solid.

In a 500-mL two-neck flask, 2-bromophenylboronic acid (6.50 g, 32.3 mmol), the crude product of **7b** (7.30 g, ~11 mmol), $Pd(PPh_3)_4$ (1.50 g, 1.30 mmol) and K_2CO_3 (11.9 g, 86.3 mmol) were dissolved in a mixture of toluene (200 mL), EtOH (50 mL), and H_2O (50 mL). The mixture was degassed by argon bubbling for 20 min. Afterwards, the solution was refluxed under argon overnight. After cooling to room temperature, water was added, and the organic phase was extracted with DCM. After washing with brine and water, the organic phases were dried over $MgSO_4$ and the solvents were removed by rotary evaporation. The residue was purified by silica gel column chromatography (hexane:DCM = 5:1 as eluent) to obtain compound **8b** as a white solid (6.51 g, 74% yield over two steps).^{1}H NMR (500 MHz, $C_2D_2Cl_4$) δ 7.46-7.34 (m, 4H), 7.16-6.71 (m, 14H), 1.57 (s, 4H), 1.16 (s, 12H), 0.57 (s, 18H). ^{13}C NMR (126 MHz, $C_2D_2Cl_4$) δ 147.09, 143.03, 141.30, 140.96, 140.59, 136.73, 136.56, 136.37, 132.43, 132.15, 131.18, 130.48, 130.05, 128.88, 128.42, 127.85, 127.37, 126.23, 125.94, 124.64, 124.44, 123.92, 74.21, 56.89, 38.05, 32.10, 31.88, 31.53. HRMS (MALDI-TOF, positive) *m/z*: Calcd. For $C_{46}H_{52}Br_2$: 762.2436; Found: 762.2430 $[M]^+$.

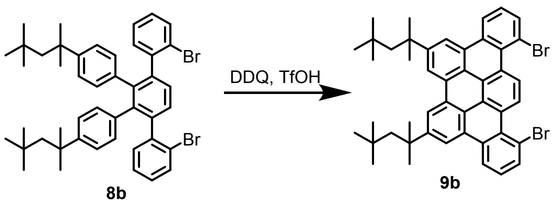

1,14-dibromo-6,9-bis(2,4,4-trimethylpentan-2-yl)tribenzo[*fg,ij,rst*]pentaphene (9b): Under an argon atmosphere, compound **8b** (800 mg, 1.09 mmol) and 2,3-dichloro-5,6-dicyano-1,4-benzoquinone (DDQ) (1.50 g, 6.61 mmol) were dissolved in 100 mL of dry DCM. The resulting solution was cooled to 0 ºC and 1.0 mL of triflic acid was added dropwise. The reaction mixture was then heated to 30 ºC and stirred for 0.5 h. The reaction was then quenched by adding methanol, and the mixture was washed three times with brine. The organic phase was dried over $MgSO_4$ and evaporated to dryness. The residue was purified by silica gel column chromatography (hexane:DCM

= 10:1 as eluent), affording **9b** as a light yellow solid (600 mg, 55% yield). ^{1}H NMR (400 MHz, CD_2Cl_2) δ 9.88 (s, 2H), 9.07 (s, 2H), 8.91 (s, 2H), 8.85 (d, J = 7.6 Hz, 2H), 8.02 (dd, J = 7.6, 1.1 Hz, 2H), 7.51 (t, J =7.6 Hz, 2H), 2.08 (s, 4H), 1.69 (s, 12H), 0.70 (s, 18H). ^{13}C NMR (101 MHz, CD_2Cl_2) δ 148.55, 135.04, 133.89, 129.49, 129.11, 128.96, 127.65, 126.49, 123.87, 123.39, 123.12, 122.40, 120.70, 120.65, 120.17, 57.26, 39.51, 32.43, 31.89, 31.77. HRMS (MALDI-TOF, positive) *m/z*: Calcd. For $C_{46}H_{46}Br_2$: 756.1966; Found: 756.1961 $[M]^+$.

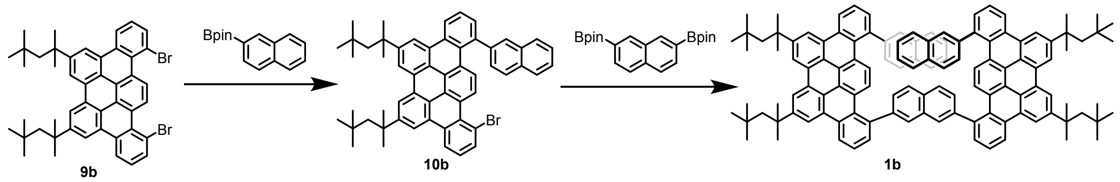

2,7-bis[14-(naphthalen-2-yl)-6,9-bis(2,4,4-trimethylpentan-2-yl)tribenzo[*fg,ij,rst*] pentaphen-1-yl]naphthalene (1b): To a 50-mL Schlenk tube, compound **9b** (190 mg, 0.251 mmol), $Pd(PPh_3)_4$ (16.0 mg, 0.0138 mmol), and K_2CO_3 (115 mg, 0.833 mmol) were dissolved in a mixture of toluene (14 mL), ethanol (4.0 mL) and H_2O (4.0 mL). After degassing the mixture by argon bubbling for 10 min and heating it to 100 °C, a toluene solution of 4,4,5,5-tetramethyl-2-(naphthalen-2-yl)-1,3,2-dioxaborolane (57.1 mg, 0.225 mmol) was added dropwise. The reaction mixture was stirred for 40 min. After cooling to a room temperature, the mixture was extracted with DCM, and the resulting organic phase was washed with brine and dried over $MgSO_4$. After removing the solvents by rotary evaporation, the residue was purified by flash silica gel column chromatography to obtain the crude 1-bromo-14-(naphthalen-2-yl)-6,9-bis(2,4,4-trimethylpentan-2-yl)tribenzo[*fg,ij,rst*]pentaphene (**10b**) as a white solid (44.1 mg, 22% crude yield). HRMS (MALDI-TOF, positive) *m/z*: Calcd. For $C_{56}H_{53}Br$: 804.3331; Found: 804.3325 $[M]^+$.

To a 25-mL Schlenk tube, the crude product of **10b** (40.1 mg, ~0.05 mmol), 2,7-bis(4,4,5,5-tetramethyl-1,3,2-dioxaborolan-2-yl)naphthalene (10.0 mg, 0.0261 mmol), $Pd(PPh_3)_4$ (5.10 mg, 0.00441 mmol) and K_2CO_3 (26.0 mg, 0.188 mmol) were added. Then the tube was evacuated and backfilled with argon three times before adding degassed THF (4.0 mL) and water (1.0 mL). The mixture was heated and stirred at 85 °C overnight. After cooling to a room temperature, the mixture was extracted with DCM, and the combined organic layers were washed with brine and dried over $MgSO_4$. After removing the solvent in vacuum, the residue was purified by silica gel column

chromatography to give **1b** as a white solid (30.1 mg, ~40% yield from crude **10b**; 9% yield over two steps). ^{1}H NMR (400 MHz, CD_2Cl_2) δ 9.10-8.74 (m, 12H), 7.92-7.03 (m, 32H), 2.12 (s, 8H), 1.73 (s, 24H), 0.73 (s, 36H). ^{13}C NMR (101 MHz, CD_2Cl_2) δ 148.34, 142.87, 141.14, 134.31, 134.07, 132.42, 132.31, 129.62, 129.47, 129.04, 128.72, 128.52, 128.35, 128.01, 127.80, 126.82, 126.62, 126.22, 126.07, 125.88, 125.65, 123.99, 122.85, 120.27, 120.10, 57.44, 39.65, 32.61, 32.12, 31.95, 31.71, 22.78, 14.01. (MALDI-TOF, positive) *m/z*: Calcd. For $C_{122}H_{112}$: 1576.8764; Found: 1576.8759 $[M]^{+}$.

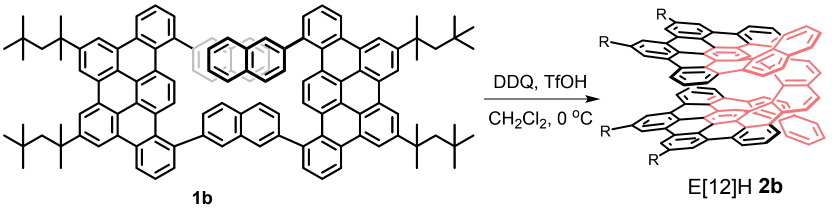

E[12]H 2b: Under argon atmosphere, compound **1b** (30.0 mg, 0.0192 mmol) and DDQ (35.5 mg, 0.156 mmol) were dissolved in dry DCM (4.0 mL). The resulting solution was cooled to 0 °C and 0.020 mL of triflic acid was added dropwise. The reaction mixture was then heated to 30 °C and stirred for 30 min. Full consumption of the starting material was indicated by thin-layer chromatography (TLC), along with the appearance of only one yellow-fluorescent product spot. The reaction was then quenched with methanol. After washing three times with brine, the organic phase was dried over $MgSO_4$ and evaporated to dryness. The residue was purified by silica gel column chromatography (hexane:DCM = 2:1 as eluent), yielding **2b** as a light yellow solid. (26.2 mg, 89% yield). ^{1}H NMR (400 MHz, CD_2Cl_2) δ 9.31 (d, J = 7.6 Hz, 2H), 9.28 (s, 2H), 9.12 (s, 2H), 8.92-8.89 (m, 4H), 8.31-8.27 (m, 4H), 8.15 (s, 2H), 7.82 (d, J = 7.6 Hz, 2H), 7.45 (d, J = 7.6 Hz, 2H), 7.17 (d, J = 7.6 Hz, 2H), 7.02-6.94 (m, 6H), 6.73 (t, J = 7.6 Hz, 2H), 6.65 (t, J = 7.6 Hz, 2H), 6.14 (d, J = 8.4 Hz, 2H), 6.03 (t, J =8.4, 2H), 2.37 (d, J = 15.0 Hz, 2H), 2.26 (d, J = 15.0 Hz, 2H), 2.04 (s, 6H), 2.02 (s, 6H), 1.89 (d, J = 15.0 Hz, 2H), 1.85 (s, 6H), 1.73 (d, J = 15.0 Hz, 2H), 1.70 (s, 6H), 0.86 (s, 18H), 0.36 (s, 18H). ^{13}C NMR (101 MHz, CD_2Cl_2) δ 148.35, 147.24, 131.57, 131.30, 130.92, 130.41, 130.32, 129.33, 128.59, 127.97, 127.52, 126.82, 126.65, 126.52, 126.19, 125.97, 125.90, 125.76, 125.23, 124.43, 124.18, 123.72, 123.62, 123.00, 122.17, 121.59, 121.23, 121.14, 120.52, 119.56, 119.48, 119.36, 119.09, 118.63, 117.98, 58.49, 57.42, 39.93, 39.48, 33.31, 33.23, 32.75, 32.34, 32.17, 31.90, 31.41, 30.26, 29.83, 22.83,

14.02. (MALDI-TOF, positive) *m/z*: Calcd. For $C_{122}H_{104}$: 1568.8138; Found: 1568.8133 $[M]^+$.

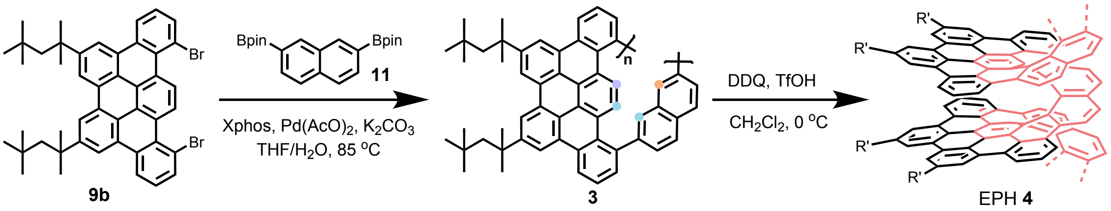

Polymer 3: In a 10-mL Schlenk tube, compound **9b** (50.2 mg, 0.0664 mmol), 2,7-bis(4,4,5,5-tetramethyl-1,3,2-dioxaborolan-2-yl)naphthalene (12.5 mg, 0.0329 mmol), $Pd(AcO)_2$ (5.10 mg, 0.0225 mmol), Xphos (20.0 mg, 0.0420 mmol) and K_2CO_3 (30.5 mg, 0.217 mmol) were dissolved in a mixture of THF (5.0 mL) and H_2O (1.0 mL). After degassing by bubbling with argon for 10 min, the mixture was heated and stirred at 100 °C for 24 h. After cooling to room temperature, the mixture was extracted with DCM, and the combined organic layers were washed with brine and dried over $MgSO_4$. After removing the solvent under the vacuum, the residue was purified by SEC (Bio-Beads S-X resins, eluent: toluene) to obtain polymer **3** as dark brown powder (32.1 mg, 65% yield).

EPH 4: Under argon atmosphere, 30.0 mg of polymer **3** and 38.0 mg of DDQ were dissolved in 5.0 mL of dry DCM, and the resulting solution was cooled to 0 ºC, followed by the dropwise addition of 0.050 mL of triflic acid. The reaction mixture was then heated to 30 ºC and stirred for 1 h. The reaction was quenched by the addition of methanol. Then, the collected solid was re-dispersed in 5.0 mL of THF and reprecipitated in methanol. The precipitates were collected via filtration, washed intensively with methanol and dried to afford EPH **4** as a black solid (25.0 mg, 82% yield).

2. Photophysical and chiroptical properties

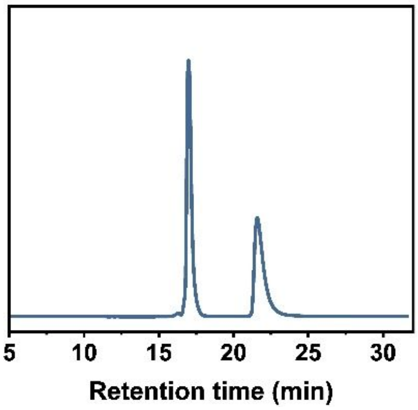

Figure S1. Chiral HPLC traces during the separation of E[12]H **2b** monitored at 370 nm (toluene/n-hexane = 3:1 used as the eluent with a flow rate of 4 mL/min).

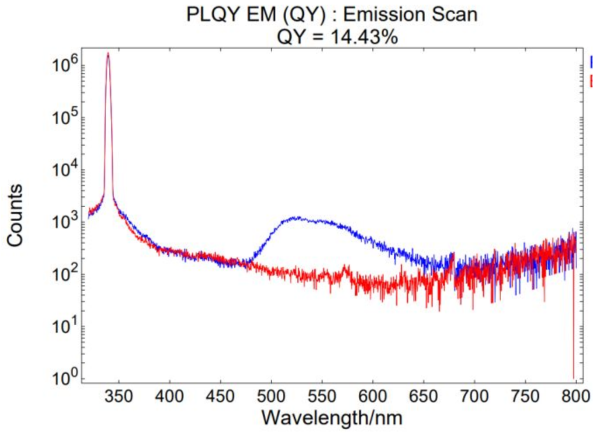

Figure S2. Absolute quantum yield measurement E[12]H **2b**. Red line: blank (DCM alone) and blue line: E[12]H **2b** in DCM at C = 10^{-5} mol/L.

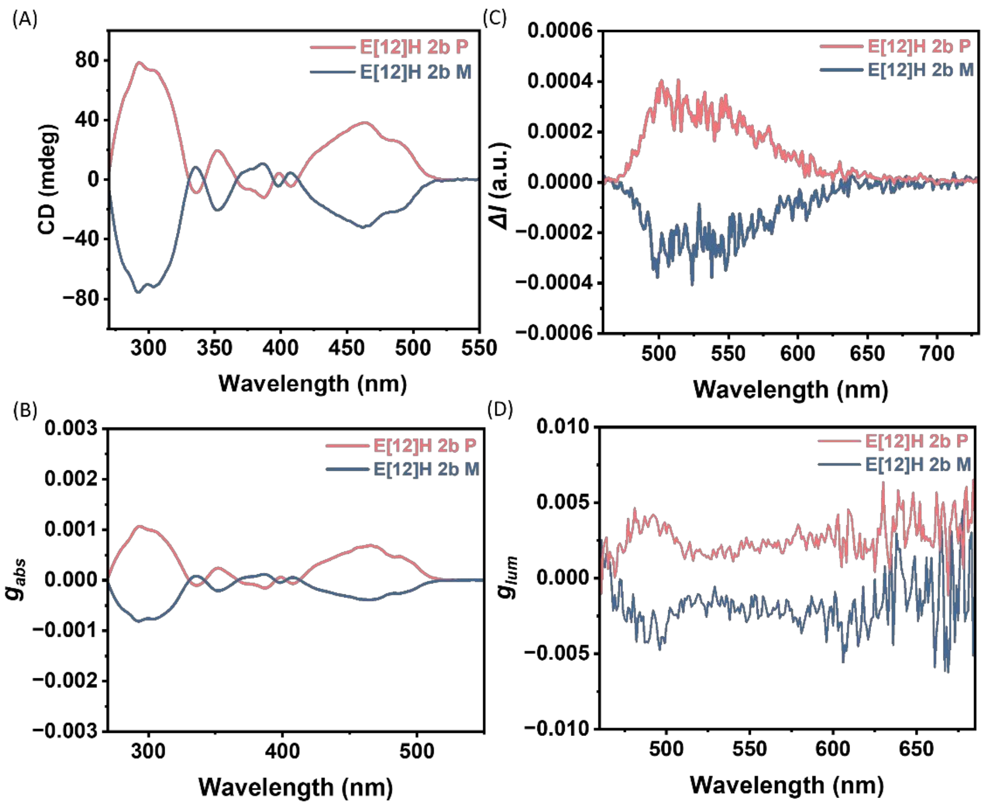

Figure S3. (A) CD spectra and (B) corresponding dissymmetry factor g_{abs} of E[12]H **2b**. (C) CPL spectra and (D) corresponding dissymmetry factor g_{lum} of E[12]H **2b** (concentration of 10^{-4} M in DCM solution).

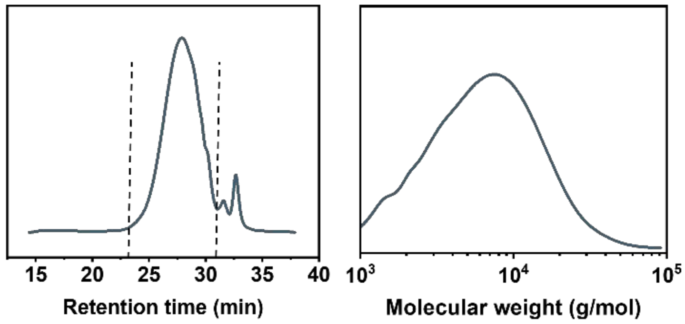

Figure S4. SEC characterization of polymer **3**. Left: SEC chart (eluent: THF, 1.0 mL/min, UV detector). Right: molecular weight distribution calculated from the selected region between the dashed lines based on the PS standard.

3. MALDI-TOF and solid-state NMR

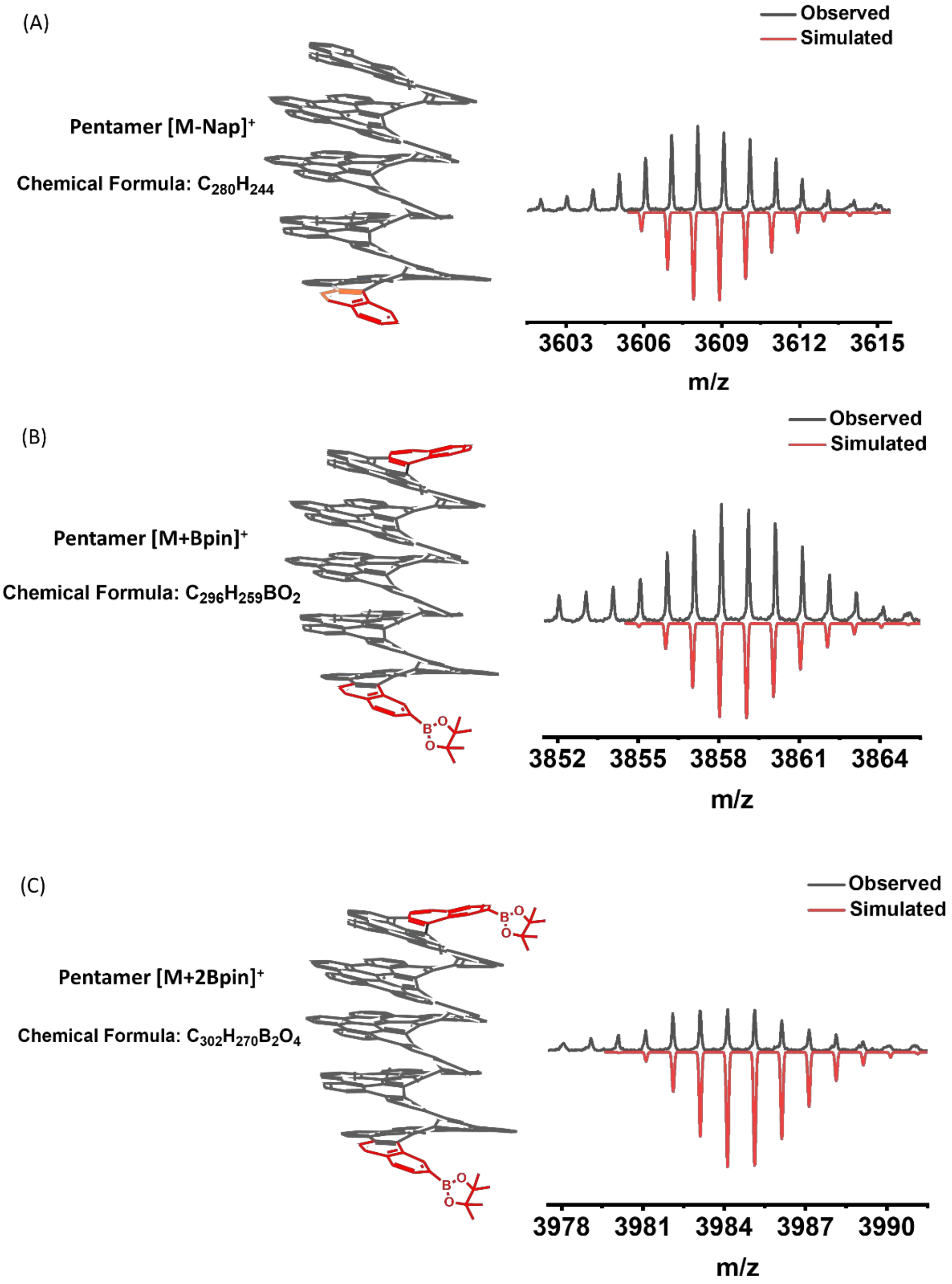

Figure S5. Assigned molecular structure for representative signals in the MALDI-TOF MS spectrum in Figure 3B, as well as their experimental and simulated isotopic distributions (reflectron mode, matrix: DCTB). (A) Pentamer [M-Nap]$^+$. (B) Pentamer [M+Bpin]$^+$. (C) Pentramer [M+2Bpin]$^+$.

The solid-state NMR measurements were performed on a Bruker AVANCE III NMR spectrometer operating at 700.25 MHz ^{1}H Larmor frequency at spinning 25 kHz MAS frequency under ambient conditions, corresponding to 40 °C sample temperature, due to frictional heating of the 2.5 mm rotor in the air bearing. The RF nutation frequency has been adjusted on the ^{1}H and the ^{13}C channels to 100 kHz, resulting in 2.5 µs 90 degree pulses. The ^{13}C CP-MAS NMR spectra have been recorded with 1 ms CP contact time and high-power swept-frequency TPPM decoupling during acquisition.

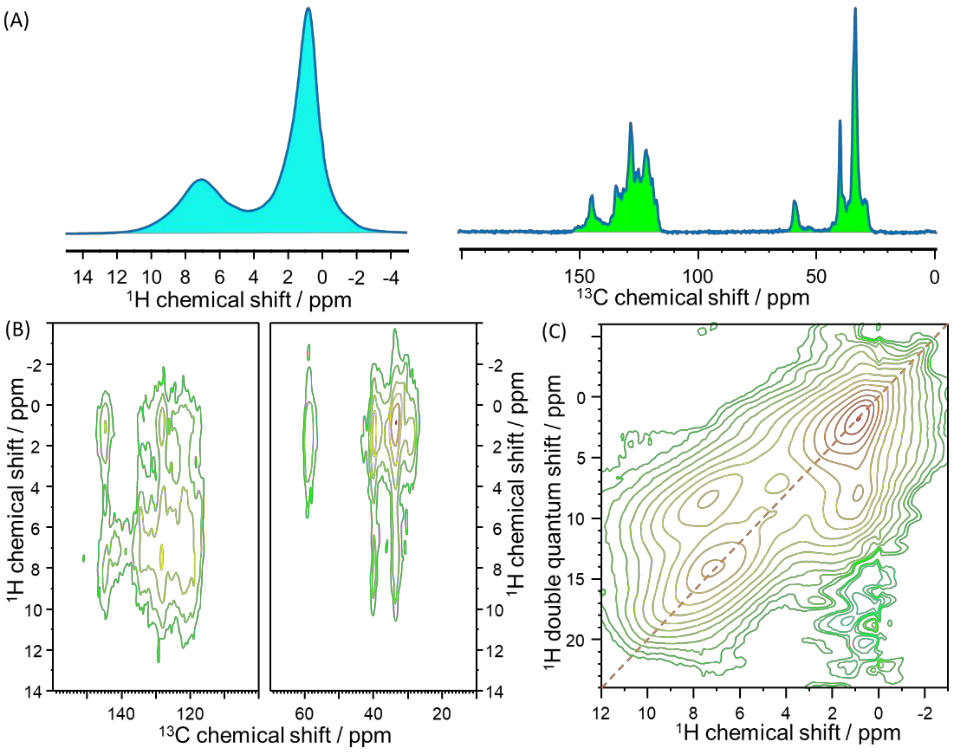

Figure S6. (A) Solid-state ^{1}H and ^{13}C CP-MAS NMR of **9b**. (B) ^{1}H-^{13}C CP-MAS correlation NMR for the **9b**, 25 kHz MAS, 700 MHz ^{1}H Larmor frequency, 1 ms CP contact, 100 kHz swfTPPM decoupling during acquisition. (C) ^{1}H DQ-SQ correlation NMR for **9b**, 25 kHz MAS, 700 MHz ^{1}H Larmor frequency, 40 µs BABA DQ excitation. Units: ppm.

Broad NMR line of EPH **4**, as seen in the ^{1}H MAS (Figure S7A and ^{13}C CP-MAS NMR spectrum (Figure 3D) indicates a significant heterogeneous broadening due to through-space π-electron shielding, which is confirmed in the DQ correlation spectrum seen as

elongation of all signals along the diagonal. Moreover, through-space contact, shown in the correlation spectra in Figure S7B-C between aromatic and aliphatic site, is significantly reduced in EPH **4,** compared to that of **9b** (Figure S6B-C), supporting the formation of EPH **4**.

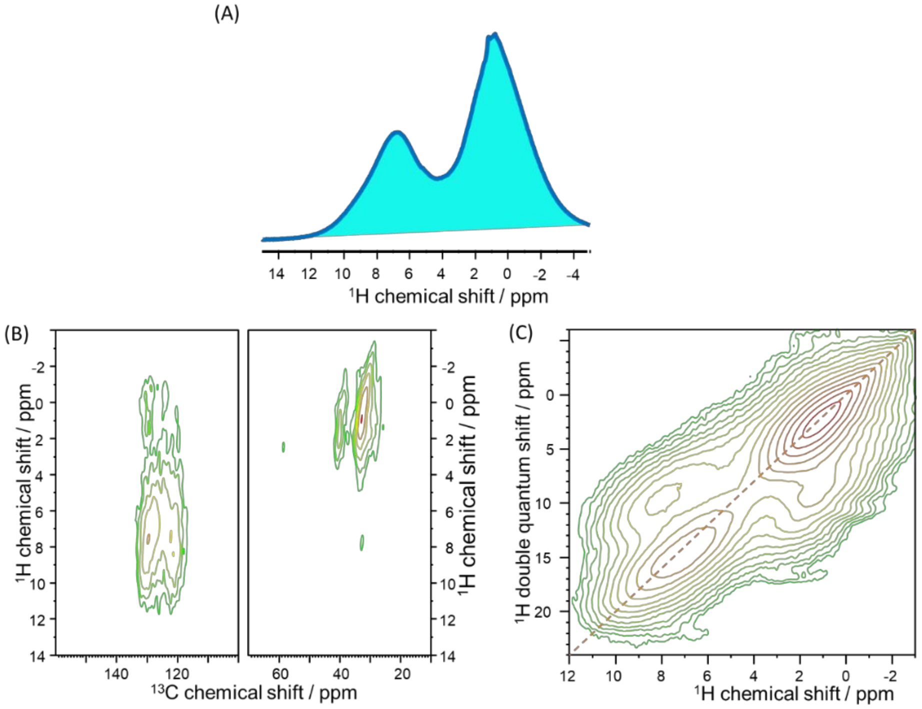

Figure S7. (A) The ^{1}H CP-MAS NMR spectrum of EPH **4**. (B) ^{1}H-^{13}C CP-MAS correlation NMR for the EPH **4**, 25 kHz MAS, 700 MHz ^{1}H Larmor frequency, 1 ms CP contact, 100 kHz swfTPPM decoupling during acquisition. (C) ^{1}H DQ-SQ correlation NMR for EPH **4**, 25 kHz MAS, 700 MHz ^{1}H Larmor frequency, 40 µs BABA DQ excitation. Units: ppm.

4. FT-IR spectrum

The comparison of the FT-IR spectra of polymer **3** and EPH **4** displays the disappearance of bands at 729, 744, 844, and 907 cm^{-1} upon the oxidative cyclodehydrogenation of **3** to EPH **4** (Figure S8), in line with previous reports on solution synthesis of graphene nanoribbons[3,4]. In particular, the intense band centered at 844 cm^{-1} is observed only for polymer **3** and assigned to the out-of-plane C-H vibration of an isolated aromatic C-H (SOLO mode)[5,6] on the naphthalene unit (marked with orange dots in Figure S8), supporting the successful formation of EPH **4**.

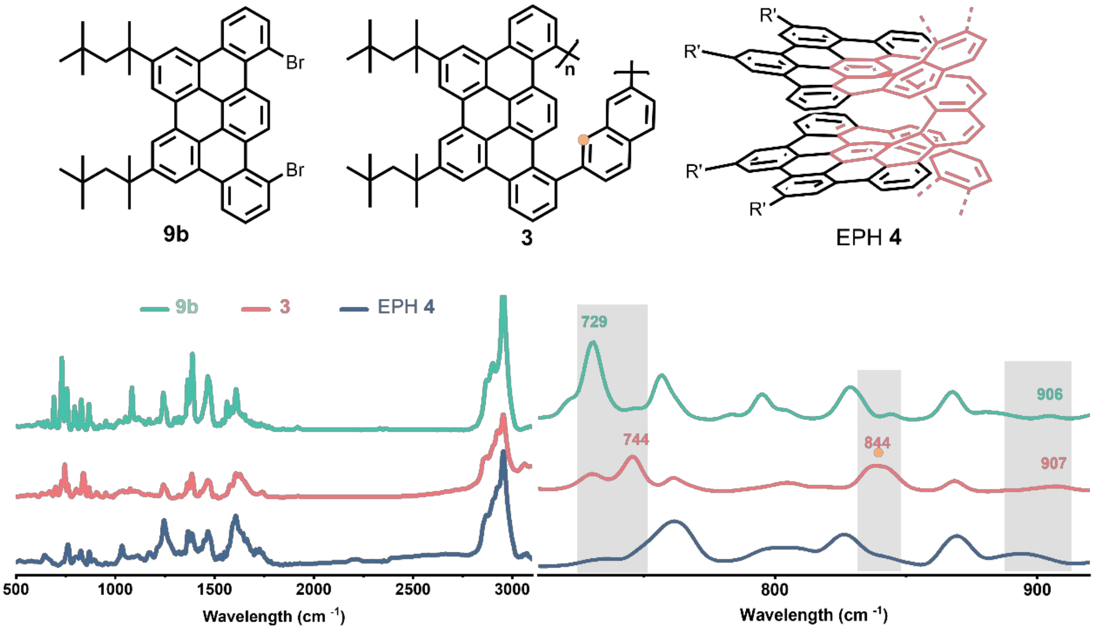

Figure S8. The FT-IR spectra of compound **9b**, polymer **3**, and EPH **4**. The proposed assignment of the SOLO band is indicated with orange dots.

5. SPM characterization

High Vacuum ElectroSpray Deposition:

HVESD was performed with molecular spray as described in previous experiments[7]. EPH **4** was dissolved in a toluene: methanol (5:1) mixture. The EPH **4** was deposited for 30 min, applying a voltage around 1.5 kV to the sample kept at room temperature.

Low Temperature AFM/STM:

Low temperature STM/AFM measurements were realized with a microscope (Omicron Nanotechnology GmbH) operated at 5 K with Nanonis RC5 electronics and based on a tuning fork sensor in the qPlus configuration (resonance frequency f = 26 KHz, at 5 K, oscillation amplitude of A = 40 pm). The STM experiments were conducted in the constant-current mode. The AFM experiments were performed in constant-height mode with CO-terminated tips.

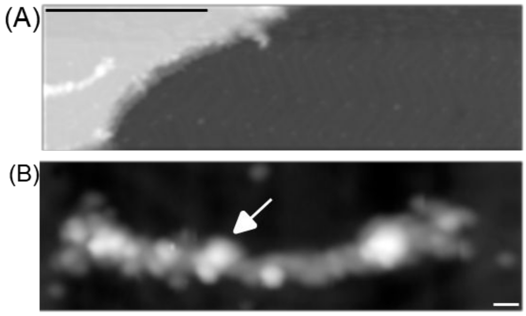

Figure S9: Electrospray deposition of EPH **4** on Au (111). (A) Large overview STM topography after HVESD. The EPH **4**s were visible on the left. (B) Zoomed STM topography on the visible EPH **4**. Scale bars: (A) = 50 nm, (B) = 2 nm.

After electrospray deposition, isolated EPH **4** was observed on the Au (111) surface. The Au (111) surface appears mostly clean, as seen in Figure S9A, where the herringbone reconstruction is visible and only kinks are filled with adsorbed species. The EPH **4** molecule is surrounded by the solvent, as indicated by the white arrow in the zoomed-in image (Figure S9B). The solvent around the EPH **4** could be removed after annealing at 100 ˚C for 10 min.

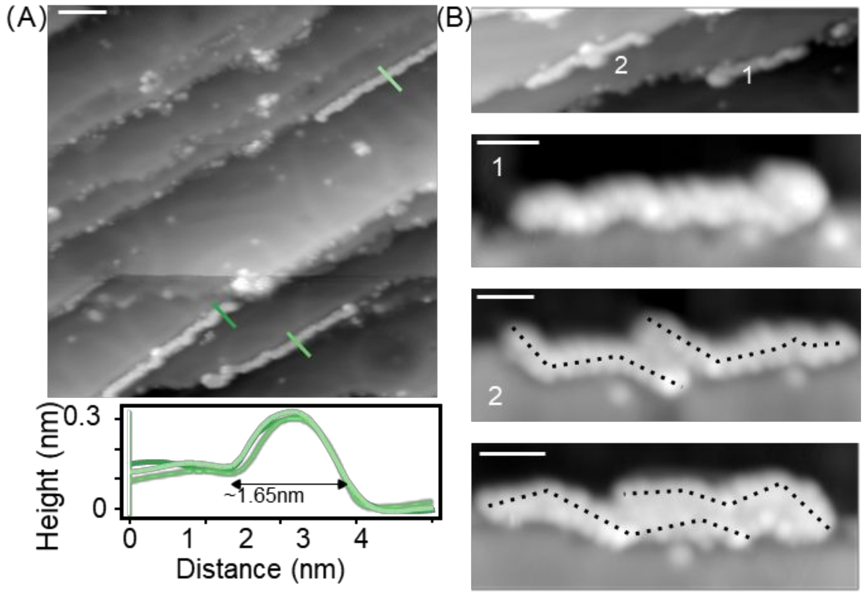

Figure S10: (A) STM topography of EPH **4** after annealing. After annealing, the EPH **4** was observed at the step edges of the surface. Profiles across the 3 places marked as EPH **4** were visible, as shown below with a width of 1.6-1.7 nm. (B) Different structures observed on the Au (111) surface showed isolated EPH **4** (1 marked), a group of two EPH **4**s (2 marked), and an assembly of the EPH **4**s from another area (bottom). Scale bars: (A) = 10 nm, (B) = 2 nm.

6. TEM characterization

Sample preparation:

Boron nitride nanotubes (BNNTs) were purchased from BNNT Materials and purified according to the reported procedure[8]. Carbon nanotubes (CNTs) were purchased from Meijo Nano Carbon Co., Ltd (MEIJO eDIPS EC2.0). Oxidative removal of the terminal caps of CNT was carried out according to the reported procedure[9]. TEM sample of EPH **4** encapsulated in BNNT was prepared as follows. A 0.5 mL dispersion of BNNT (0.01 mg/mL) in methanol was mixed with 0.09 mg of EPH **4** dissolved in 0.4 mL of toluene. After sonication for 1 min in a bath sonicator, the mixture was heated for 1 h at 80 °C. The resulting mixture was sonicated again for 30 sec, and the dispersion was dropped onto a TEM microgrid (RO-C15, Okenshoji Co. Ltd.) in three portions of 20 μL each. The TEM grid was then dried in a vacuum (4 hPa) for 30 min. A TEM sample of EPH **4** encapsulated in CNT was prepared similarly.

TEM observation:

TEM observation was carried out on an aberration-corrected TEM instrument (FEI Titan Cubed) at an acceleration voltage of 80 kV under 4×10^{-6} Pa in the specimen column using a monochromator for the incident electron beam (ΔE = 0.15 eV). Images were captured and processed on a CMOS camera (Gatan OneView, in situ mode, 4096×4096 pixels) operated in the binning 2 mode. The images were recorded at under-focus conditions (defocus value: ca 10 nm). Images were recorded with the exposure time of 1.0 s at an electron dose rate of about 1×10^{6} e^{-} nm^{-2} s^{-1}. Note that the image of BNNT was vibrating throughout the observation[10], causing a decrease in spatial resolution. Experimental TEM images were analyzed and processed on DigitalMicrograph software (Gatan, Inc.). TEM image simulation was performed by using a multi-slice procedure implemented in Elbis software[11] using the experimental observation conditions described above.

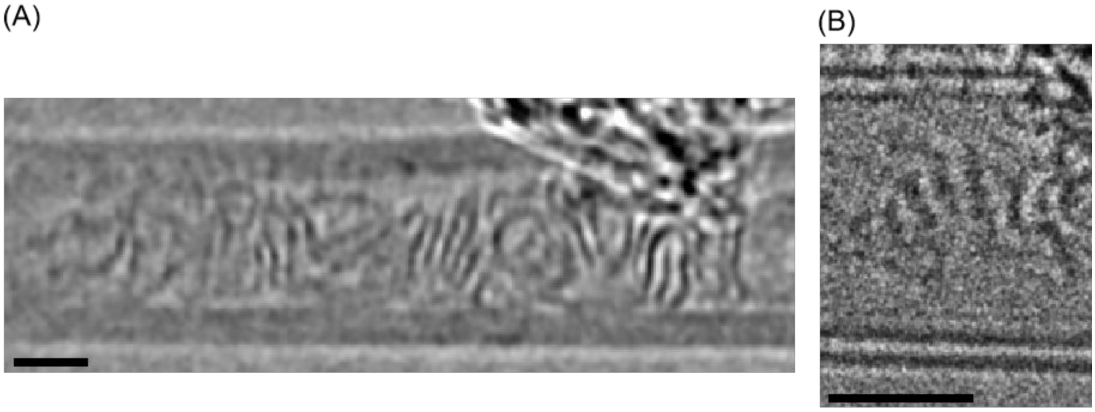

Figure S11: (A) HR-TEM image of EPH **4** in a double-walled BNNT. The wider field of view image of Figure 4E. (B) HR-TEM image of EPH **4** in a double-walled carbon nanotube. Scale bar: 2 nm.

7. DFT calculations

The density functional theory (DFT) calculations were performed using the Gaussian 16 C.01 version program package[12] with B3LYP/6-31G(d) level[13]. Transition state calculations employ the built-in TS algorithm within the Gaussian program and compute transition state frequencies to ensure that the structure possesses exactly one imaginary frequency.

The TD-DFT calculations were performed at the PBE0/def2-TZVP level in the ORCA program[14]. The molecular orbitals and spin density distribution were visualized using the VMD 1.9.3 program[15] and Multiwfn 3.8[16]. The g value of S_0 (g_{abs}) and S_1 (g_{lum}) configurations in E[12]H **2b** were calculated by the equation:

$$g_{abs}/g_{lum} = 4\cdot\cos\theta\cdot|\mu|/|m|$$

where the g_{abs}/g_{lum} is the g values in adsorption and luminescence spectra, the $|\mu|$, $|m|$, and θ are the electric transition dipole moment, magnetic transition dipole moment, and the angle between the electric and magnetic transition dipole moments. The alkyl groups in all calculation were removed to simplify the calculations

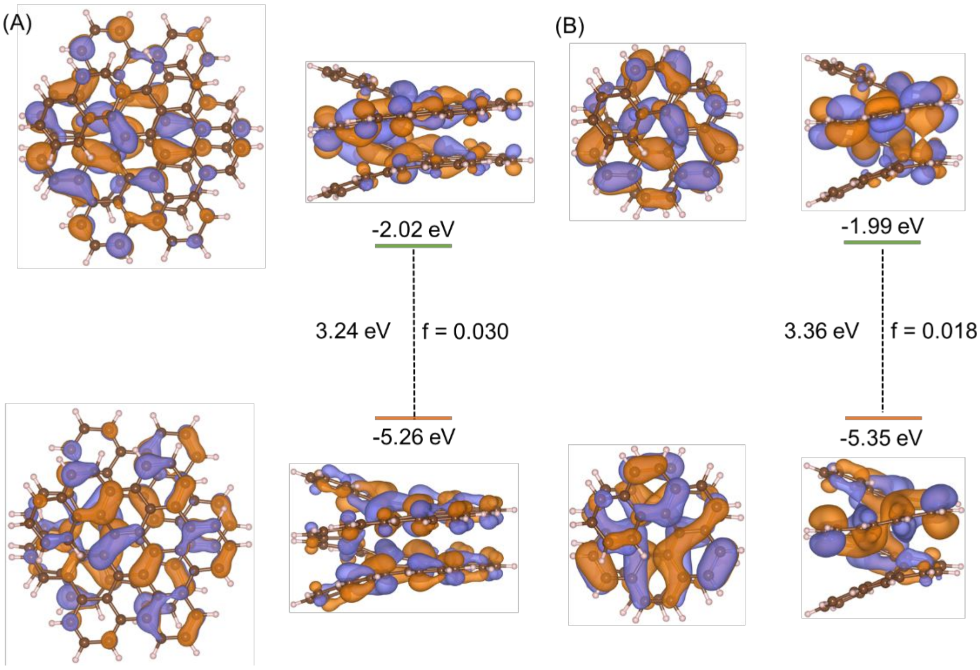

Figure S12. Frontier molecular orbitals and $S_0 \rightarrow S_1$ oscillator strengths (f) of (A) E[12]H **2b** and (B) non-extended [12]helicene in the ground state.

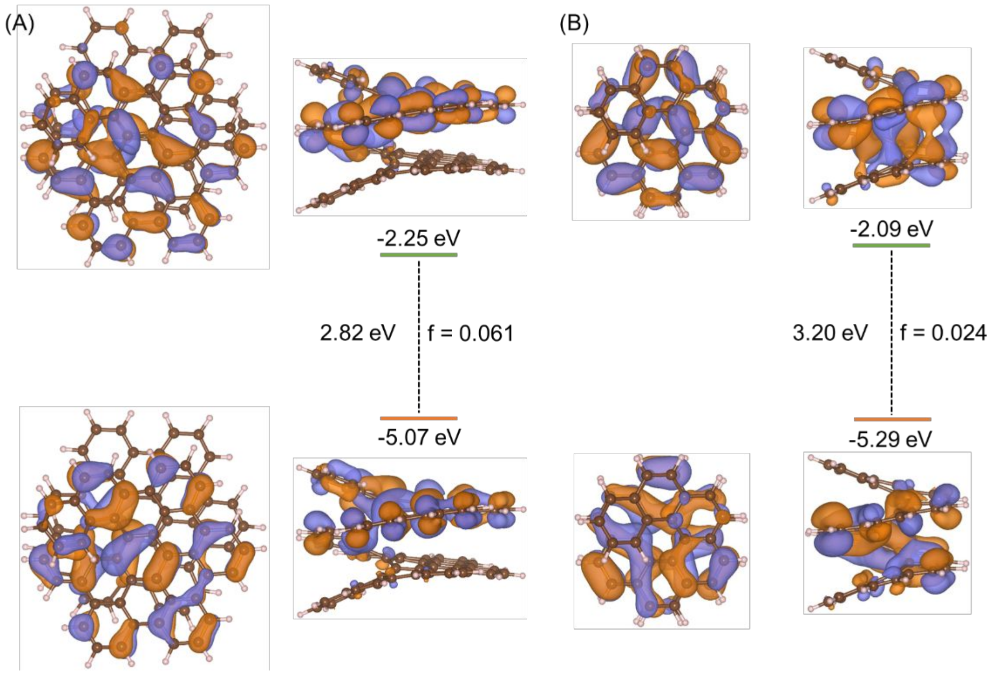

Figure S13. Frontier molecular orbitals and $S_1 \rightarrow S_0$ oscillator strengths (*f*) of (A) E[12]H **2b** and (B) non-extended [12]helicene in the excited state.

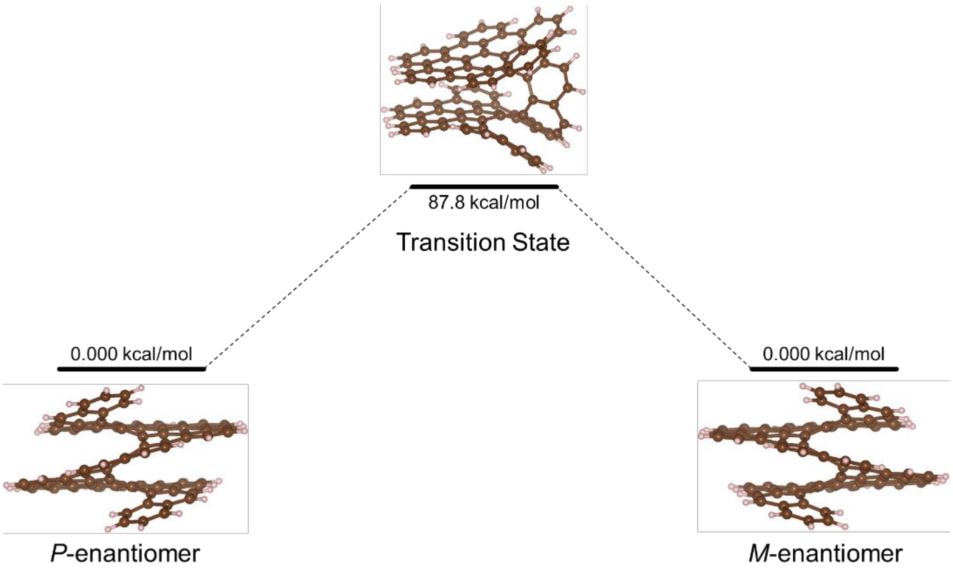

Figure S14. *P*/*M* isomerization barrier of E[12]H **2b**. The relative Gibbs free energies were calculated at the B3LYP/6-31G(d) level.

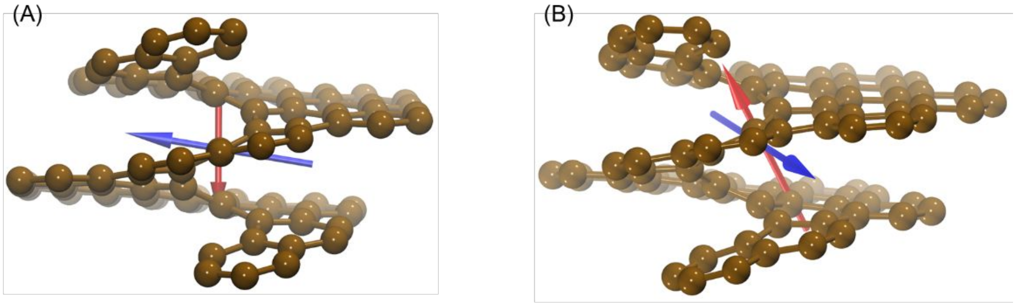

Figure S15. Transition dipole moments of E[12]H **2b** for (A) $S_0 \rightarrow S_1$ and (B) $S_1 \rightarrow S_0$ transition. The electric transition dipole moments (μ) are shown in blue, and the magnetic transition dipole moments (m) are shown in red. The length of the m vector is amplified 200 times for clarity.

The E[12]H and the corresponding infinite polyhelicene (PH) and EPH have been investigated from first-principle by employing a supercell implementation of Density-Functional Theory (DFT) based on plane waves and pseudopotentials, as available in the Quantum ESPRESSO package[12]. Calculations were performed by using PBE exchange correlation (xc) functional and ultrasoft pseudopotentials from the SSSP library (PBE Precision v1.3.0)[13], with a cutoff energy for the wavefunctions (density) of 48 (480) Ry. Dispersion corrections were included in an optimized vdW-DF-like scheme[14]. To mimic isolated E[12]H, PH and EHP with periodic boundary conditions, a vacuum region of about 12 Å was added in the non-periodic directions to avoid fictitious interactions with the system replicas. The atomic positions within the cell were fully relaxed until forces were less than 10^{-4} Ry/bohr. For PH and EPH, variable cell calculations were performed with a convergence threshold for the pressure of 0.1 kbar.

Figure S12A reports the structural models of E[12]H as resulting from DFT structural optimization, where *t*Bu groups are used for simplicity. We find that torsion angles (a-b-c-d and d-e-f-g, as defined in the main text), distance between the two nanographene planes, and their nearly AA stacking are in good agreement with data obtained from X-ray crystallography (see Figures 2 and S13-14). The lateral extension is also in

agreement with STM profiles (see Figure S10). The π -conjugated nature of the system is evident from the plot of the frontier orbitals (H and L/L+1), whereas the extension of interlayer π-π interaction can be visualized by using the intramolecular non-covalent interactions (NCI) analysis[15], both shown in panel B.

The periodic structures, used to study band dispersion and compute the effective mass *m**, are reported in Figure S12C, D. Panel C shows the structure of infinite PH, which has been built by imposing $L6_1$ symmetry, i.e., a six-fold rotation of the C_4H_2 repeated unit accompanied by a translation. This PH structure represents the minimal periodic structure with rational roto-translation parameters closest in energy to the global energy minimum structure[16]. As recently demonstrated by Porsev et al.[16], the minimal energy structure is *over-twisted* with respect to the ideal $L6_1$ structure, with a rotation angle of 61.43° instead of 60.00°. Comparing the top views of E[12]H and PH (Figure S12A,C), one can clearly appreciate that the rotation angle for the helicene structure within E[12]H (blue atoms) is also larger than 60°, with the atoms of different layers not being perfectly superimposed.

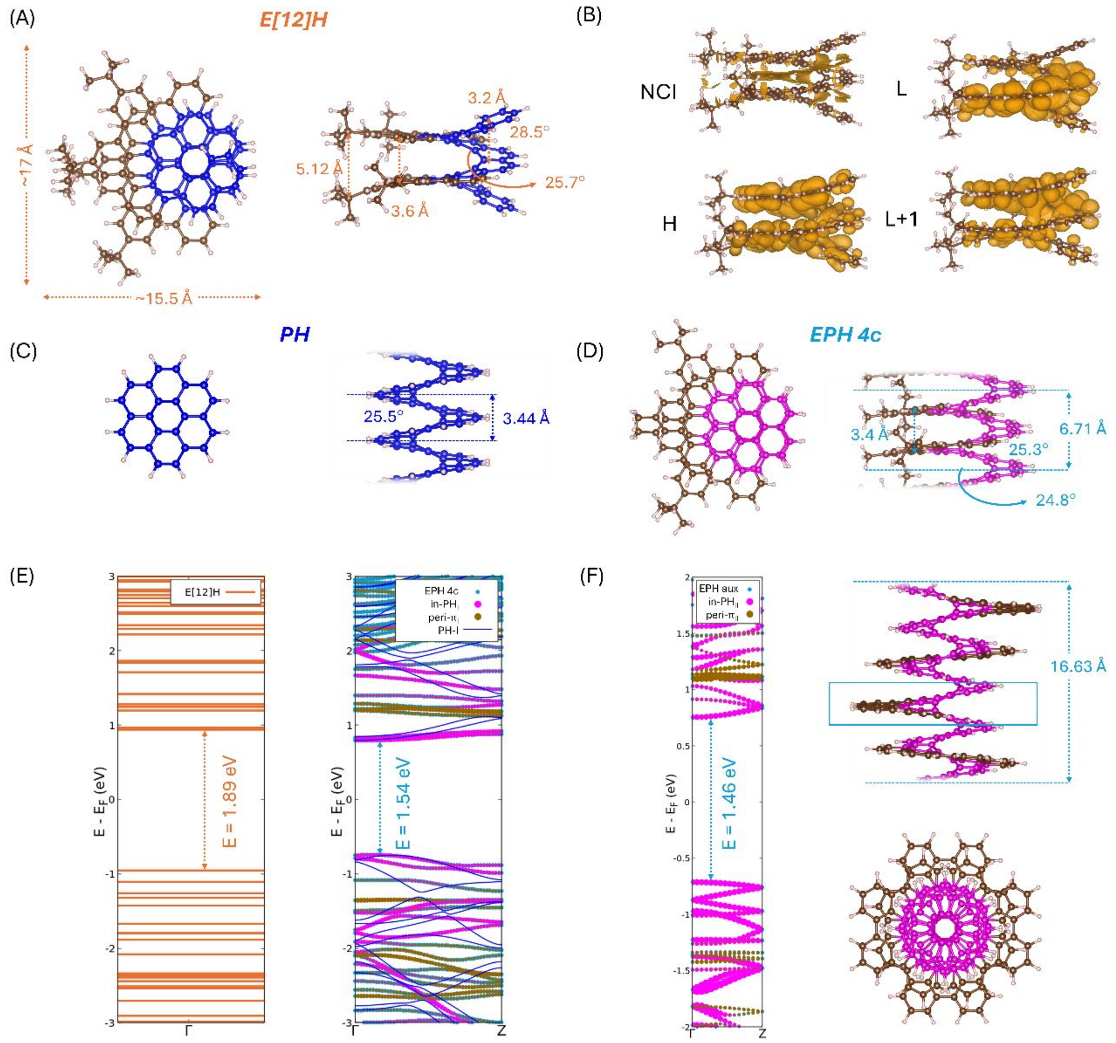

Figure S16. (A, C, D) Ball-and-stick models for the DFT optimized atomic structures of E[12] H, PH and EPH **4c**, as described in the text (C and H atoms are in brown and white, respectively; the C atoms of PH and of the helicene embedded in E[12] H and EPH **4c** are highlighted with a different color for clarity). (B) Non-covalent interactions (NCI) analysis and frontier orbitals (HOMO, H, and LUMO/LUMO+1, L/L+1) of E[12] H. (E) Band structures of E[12]H and EPH **4c**. The bands projected on the C atoms of the internal PH (in-PH_I, magenta dots in panel D) and on the remaining C atoms of the nearly co-facial peripheral π-conjugated regions (peri-π_I, brown dots in panel D) are also plotted for EPH **4c**. Band structure of PH-I (computed in the same unit cell as EPH **4c**) is also displayed. (F) Ball-and-stick model and band structure of an auxiliary EPH (EPH aux). The roto-translational unit is highlighted by the box. The internal PH (in-PH_{II}) is highlighted by magenta-colored C atoms. Bands projected on in-PH_{II} and the peripheral π-conjugated regions (peri-π_{II}, brown dots) are also plotted.

In order to model the corresponding infinite EPH structure, one would thus need an extremely long supercell able to properly approximate the real irrational roto-

translation, considering that the unit to rotate is the entire molecule and not only the C_4H_2 monomer as for PH. Moreover, we cannot resort in this case to a minimal, under-twisted model as we did for PH. In fact, if we try to just repeat the E[12]H structure periodically (see the model EPH **4c** structure in Figure S12D, where the alkyl groups are substituted by methyl groups to reduce the steric hindrance and avoid non-physical distortions), we end up with an infinite structure with broken rotational symmetry due to the presence of the peripheral π-conjugated regions. Despite the broken symmetry, the optimized pitch for the inner helical core of EPH **4c** is basically the same as that of PH (~3.44 Å), and only slightly larger than for E[12]H (~3.21 Å, see Figure S12A). And the same holds for the nanographene interlayer distance, the dihedral angles and the nearly-AA stacking, which are only slightly modified relative to E[12]H. The extension of the interlayer π-π interaction (not shown here) is also very similar to that of E[12]H (Figure S12B).

Whereas the structural properties are approximately the same, the electronic properties display non-negligible differences. Panel E reports the band structures of E[12]H and EPH **4c**. In addition to the gap reduction (from 1.89 eV for E[12]H to 1.54 eV for EPH **4c**), the bands of the EPH periodic structure (light blue dots) display a non-negligible dispersion. To better understand its origin, the bands are projected on the C atoms of the internal polyhelicene (here labelled in-PH_I, magenta dots with size proportional to the k-resolved projection) and on the remaining C atoms of the nearly co-facial peripheral π-conjugated regions (here labelled peri-π_I, brown dots, with size proportional to the k-resolved projection). Superimposing the bands of PH computed in the same unit cell of EPH **4c** (PH-I, solid blue lines, where the top of the valence and bottom of the conductions are aligned with those of EPH **4c**), we can clearly see that they display a pattern reminiscent of the projected bands due to the in-PH_I portion of EPH **4c** (magenta dots). These bands are, however, interlaced with those of the peri-π_I regions in EPH **4c**, indeed less dispersive. These interpositions, connected to the broken helical symmetry, result in a reduced dispersion, and in a corresponding increase of the effective mass m^*. Indeed, the latter amounts to 0.52 m_0 in PH-I, while it is doubled in EPH **4c**, thus showing how sensitive the band structure is to the helix pitch, distortions and symmetry breaking. This picture is confirmed by looking at the band structure of another auxiliary EPH, which we built by considering the single extended layer as a repeated unit and introducing one additional hexagon along the helix to have a fully roto-translated system of affordable size. Here, the frontier bands are fully localized on

the inner polyhelicene (in-PH_{II}, magenta dots), while the bands related to the peripheral π-conjugated moieties (peri-π_{II}) are further in energy. The effective mass is $m^* = 0.3$ m_0, unchanged with respect to that of the corresponding isolated PH-II (not shown) with the same roto-translational parameters.

As can be seen in the model reconstructed from TEM images (Figure 4G), the real structures apparently maintain the helical symmetry and are likely to be more similar the ideal PH also in terms of dispersion. For this reason and based on the discussion above, we approximate the effective mass with that computed for PH-I and II, i.e. $m^* = 0.4 \pm 0.1$ m_0.

.

8. Terahertz spectroscopy characterization

We implemented an optical pump–terahertz probe (OPTP) setup utilizing a commercial Ti:sapphire laser system. This laser operated at a repetition rate of 1000 Hz, emitted pulses approximately 50 fs in duration, and was centered at a wavelength of 800 nm. The output beam was split into three separate paths dedicated to generating THz radiation, delivering the optical excitation, and performing electro-optic sampling. THz pulses were generated via optical rectification in a ZnTe crystal. The detection of the transmitted THz field was accomplished using free-space electro-optic sampling with a second ZnTe crystal. To analyze the time-dependent THz photoconductivity response, we varied the time delay between the optical pump and sampling pulses using an automated translation stage, recording changes in THz transmission at the peak electric field. All experiments were carried out at ambient temperature in an environment purged with dry nitrogen.

Drude-Smith model

We fitted the frequency-resolved photoconductivity by the Drude-Smith (DS) model[17,18]. The model was used to phenomenologically describe the charge transport properties in nanostructured semiconductors[19] following:

$$\sigma(\omega) = \frac{\omega_{\mathrm{p}}^2 \varepsilon_0 \tau_{\mathrm{DS}}}{1 - i\omega\tau_{\mathrm{DS}}}\left(1 + \frac{c}{1 - i\omega\tau_{\mathrm{DS}}}\right)$$

where τ_{DS}, ω_p, ε_0 represented the charge scattering time, the plasma frequency, and vacuum permittivity, respectively. Here the parameter c was used to parameterize the backscattering probability ranging from 0 (isotropic scattering, equivalent to Drude model) to -1 (100% backscattering).

9. X-Ray single crystallography

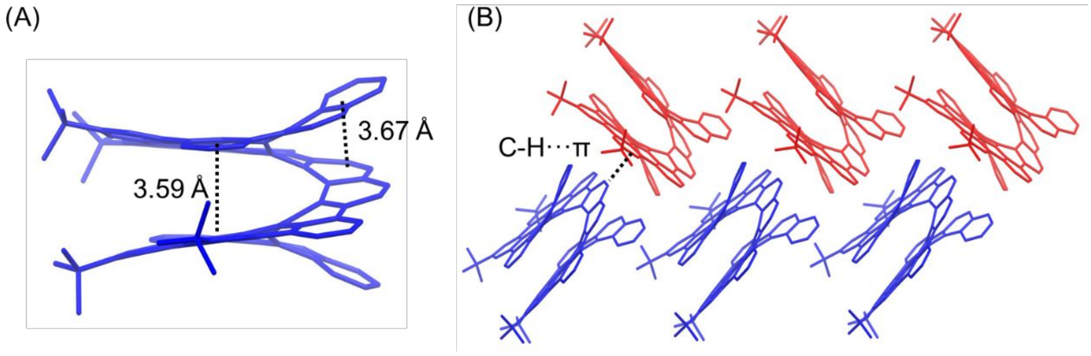

Figure S17. The single crystal and molecular packing of E[12]H **2a**.

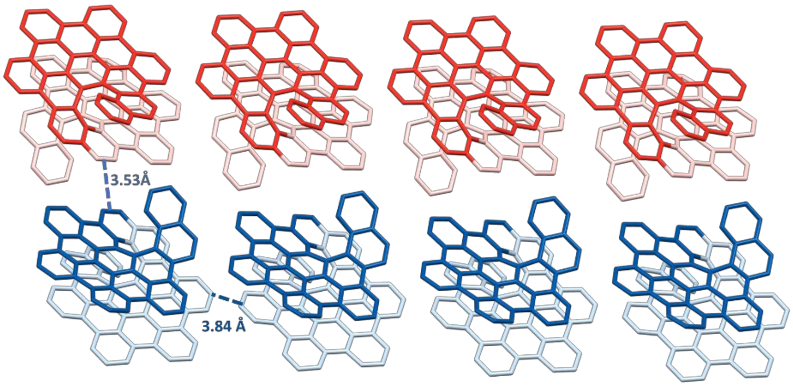

Figure S18. The single crystal and molecular packing of E[12]H **2b**. All hydrogen atoms and the alkyl chains are omitted for clarity.

Table S1. Crystal data and structure refinement for E[12]H **2b**

Identification code	E[12]H **2b**	
Empirical formula	$C_{122}H_{104}$ + [solvent]	
moiety formula	$C_{122}H_{104}$ + [solvent]	
Formula weight	1570.05	
Temperature	120(2) K	
Wavelength, radiation type	1.54186Å, CuKα	
Diffractometer	STOE STADIVARI	
Crystal system	Triclinic	
Space group name, number	P -1, (2)	
Unit cell dimensions	a = 12.3489(3) Å	α = 102.071(2)°
	b = 16.9091(4) Å	β = 100.824(2)°
	c = 25.0023(6) Å	γ = 105.318(2)°
Volume	4757.6(2) $Å^3$	
Number of reflections	44901	
and range used for lattice parameters	4.62° <= θ<= 69.51°	
Z	2	
Density (calculated)	1.096 Mg/m^3	
Absorption coefficient	0.464 mm^{-1}	
Absorption correction	None	
F(000)	1672	
Crystal size, colour and form	0.250 x 0.250 x 0.330 mm^3, red block	
Theta range for data collection	4.622 to 67.995°.	
Index ranges	-13<=h<=14, -20<=k<=20, -29<=l<=29	
Number of reflections:		
collected	50884	
independent	16186 [R(int) = 0.0177]	
observed [I>2sigma(I)]	14939	
Completeness to theta = 67.7°	93.6 %	
Refinement method	Full-matrix least-squares on F^2	
Data / restraints / parameters	16186 / 150 / 1188	
Goodness-of-fit on F^2	1.050	
Final R indices [I>2sigma(I)]	R1 = 0.0712, wR2 = 0.1963	
R indices (all data)	R1 = 0.0743, wR2 = 0.1992	
Largest diff. peak and hole	1.053 and -0.556 eÅ	

Table S2. Crystal data and structure refinement for E[12]H **2a**

Identification code	E[12]H **2a**
Empirical formula	$C_{106}H_{72}$
moiety formula	$C_{106}H_{72}$
Formula weight	1345.63
Temperature	120(2) K
Wavelength, radiation type	0.71073Å, MoKα
Diffractometer	STOE IPDS 2T
Crystal system	Monoclinic
Space group name, number	P 2_1/n, (14)
Unit cell dimensions	a = 19.5617(10) Å α = 90°
	b = 12.9309(5) Å β = 101.593(4)°
	c = 28.5811(16) Å γ = 90°
Volume	7082.1(6) $Å^3$
Number of reflections	18225
and range used for lattice parameters	2.26° <=θ<= 28.32°
Z	4
Density (calculated)	1.262 Mg/m^3
Absorption coefficient	0.071 mm^{-1}
Absorption correction	None
F(000)	2832
Crystal size, colour and form	0.270 x 0.080 x 0.060 mm^3, brown
block	
Theta range for data collection	2.259 to 27.931°.
Index ranges	-24<=h<=25, -16<=k<=16, -
37<=l<=37	
Number of reflections:	
collected	36038
independent	16788 [R(int) = 0.0652]
observed [I>2sigma(I)]	9095
Completeness to theta = 25.2°	99.7 %
Refinement method	Full-matrix least-squares on F^2
Data / restraints / parameters	16788 / 184 / 1058
Goodness-of-fit on F^2	1.173
Final R indices [I>2sigma(I)]	R1 = 0.1219, wR2 = 0.2111
R indices (all data)	R1 = 0.2124, wR2 = 0.2552
Largest diff. peak and hole	0.337 and -0.334 $eÅ^{-3}$

Table S3. Overview of carrier mobilities in graphene nanoribbons measured by THz spectroscopy.

Sample	m^* ($\times m_0$)	c	τ_{DS} (fs)	μ ($cm^2\ V^{-1}\ s^{-1}$)	μ_{dc} ($cm^2\ V^{-1}\ s^{-1}$)	Ref.
**EPH 4**	0.4 ± 0.1	–0.81 ± 0.01	16 ± 2	70 ± 20	13 ± 4	This work
**6-CZGNR-(2,1)**	0.085	–0.97	29 ± 2	600 ± 41	18 ± 1	S25
**(4,2)-CcGNR**	0.22	–0.96	43 ± 2	343 ± 16	13 ± 1	S26
**(6,2)-CcGNR**	0.23	–0.96	30 ± 2	229 ± 15	9 ± 1	
**GNR-OMe**	0.097	–0.92 ± 0.01	27 ± 7	60 ± 16	5 ± 1	S27
**cMGNR**	0.8	–0.97 ± 0.01	36 ± 2	79 ± 4	2.4 ± 0.1	S28
**PyGNR**	0.55	–0.97	38 ± 1	121 ± 3	3.6 ± 0.1	S29
**FGNR**	0.48	–0.99 ± 0.01	28 ± 1	102 ± 4	1 ± 1	S30
**PAL 1**	0.3	–0.96 ± 0.01	30 ± 3	176 ± 17	7 ± 2	S31
**PAL 2**	0.72	–0.96 ± 0.01	30 ± 3	73 ± 7	3 ± 1	

10. ^{1}H and ^{13}C NMR spectra

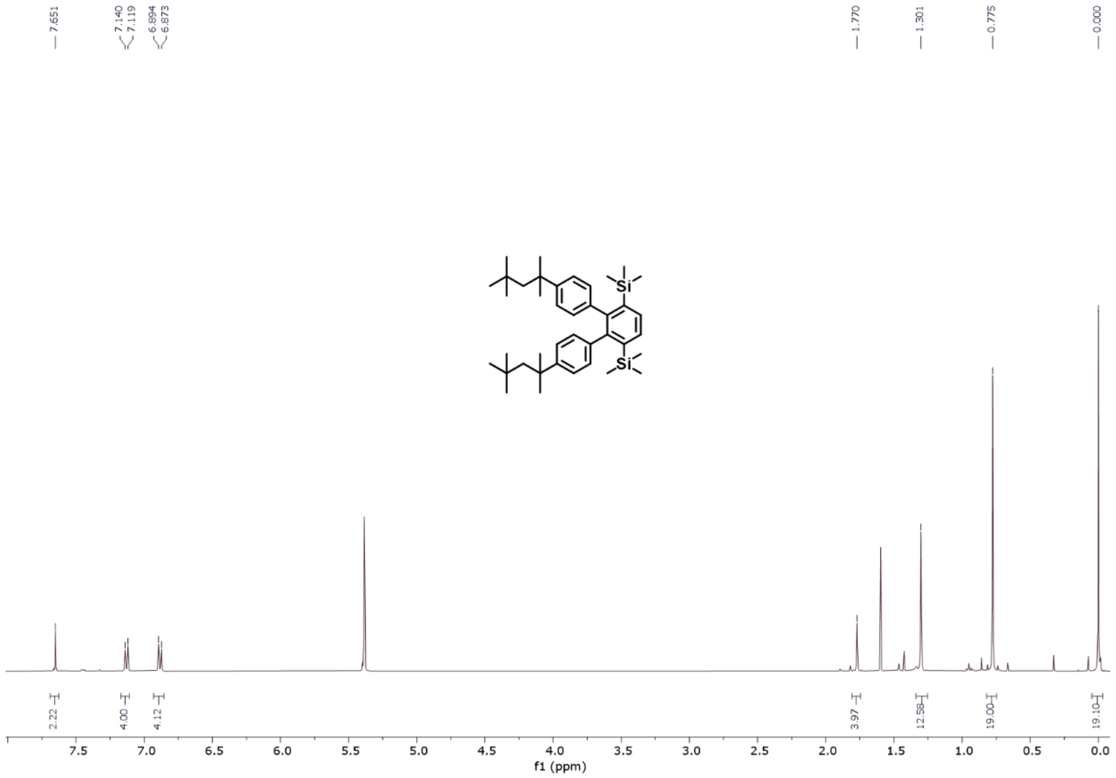

Figure S19. ^{1}H NMR spectrum of compound **6b** in CD_2Cl_2 (400 MHz).

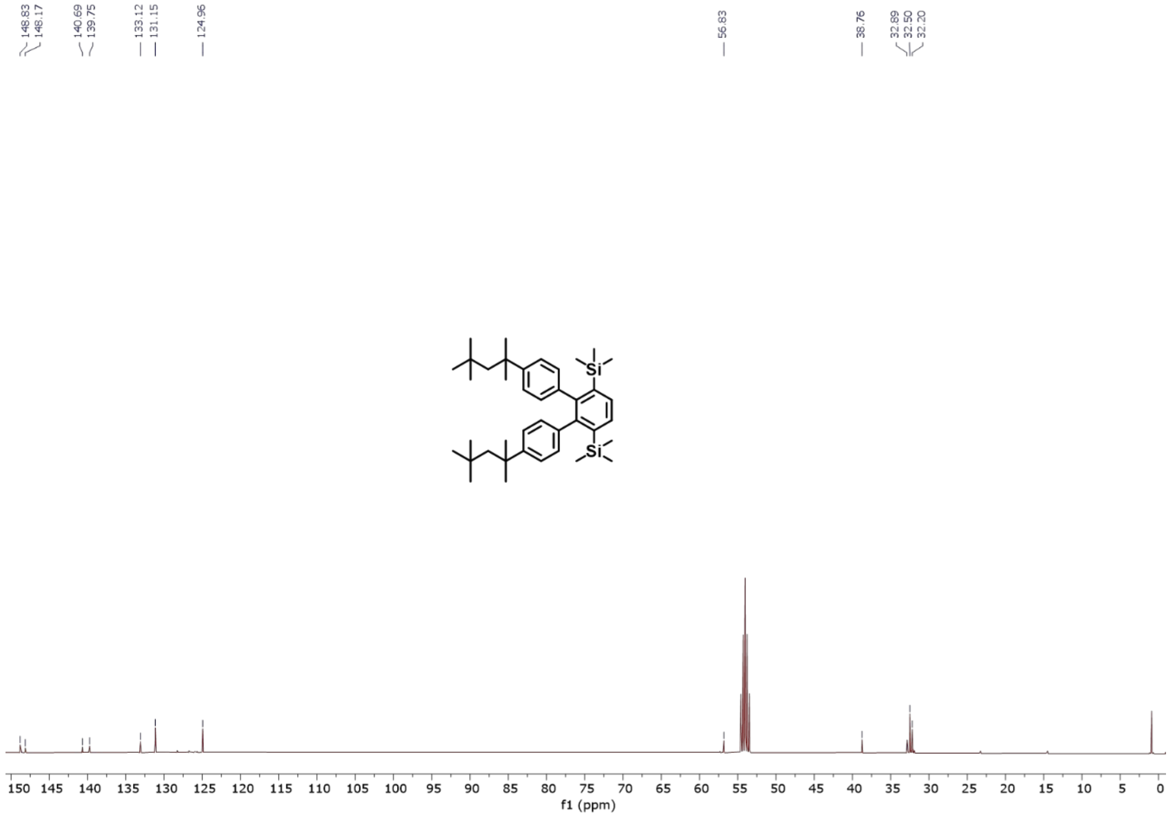

Figure S20. ^{13}C NMR spectrum of compound **6b** in CD_2Cl_2 (101 MHz).

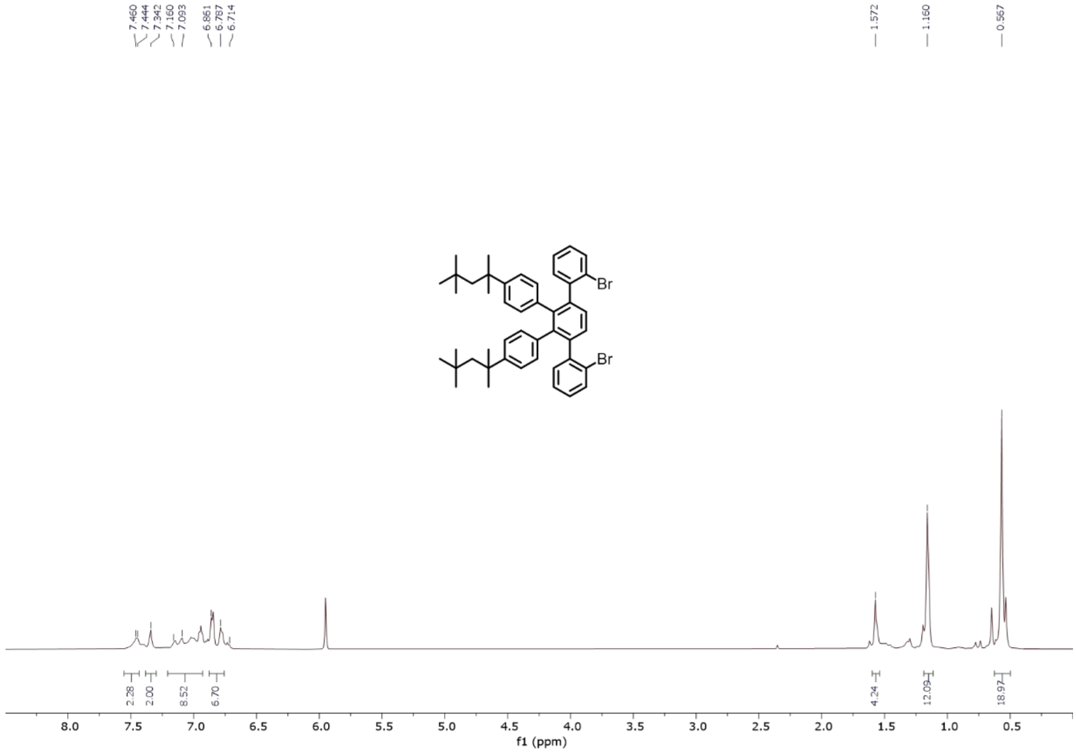

Figure S21. ^{1}H NMR spectrum of compound **8b** in $C_2D_2Cl_4$ (500 MHz) at 373K.

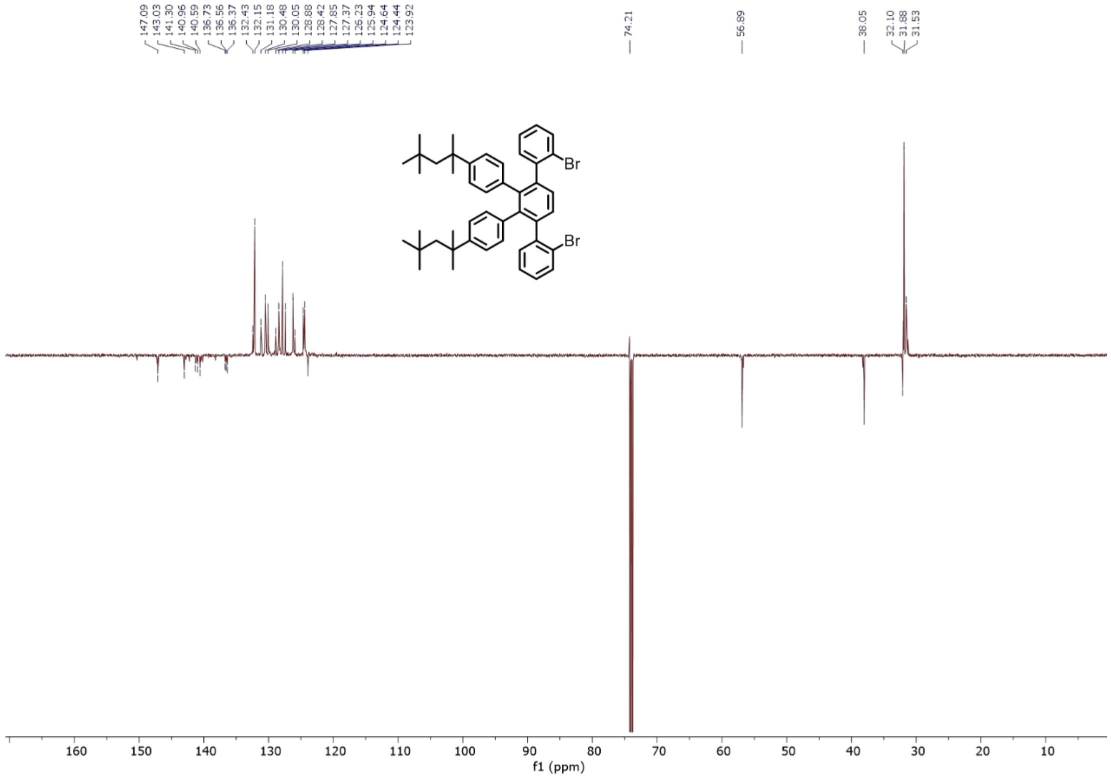

Figure S22. ^{13}C NMR spectrum of compound **8b** in $C_2D_2Cl_4$ (126 MHz) at 373K.

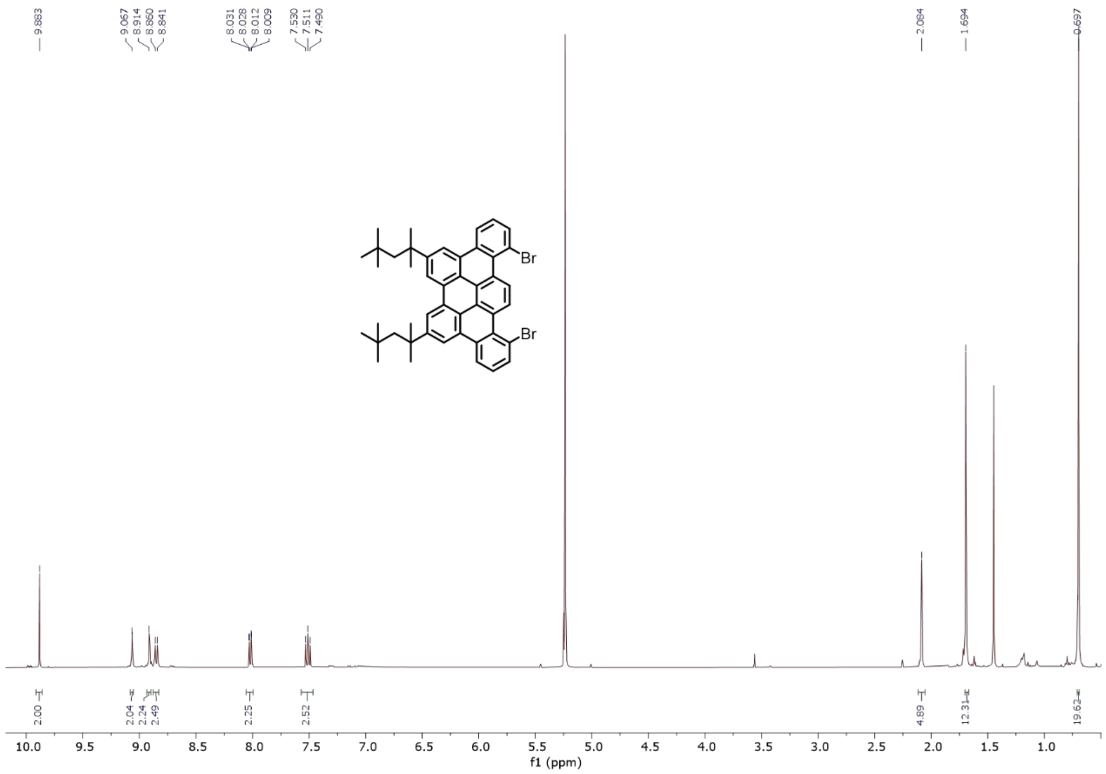

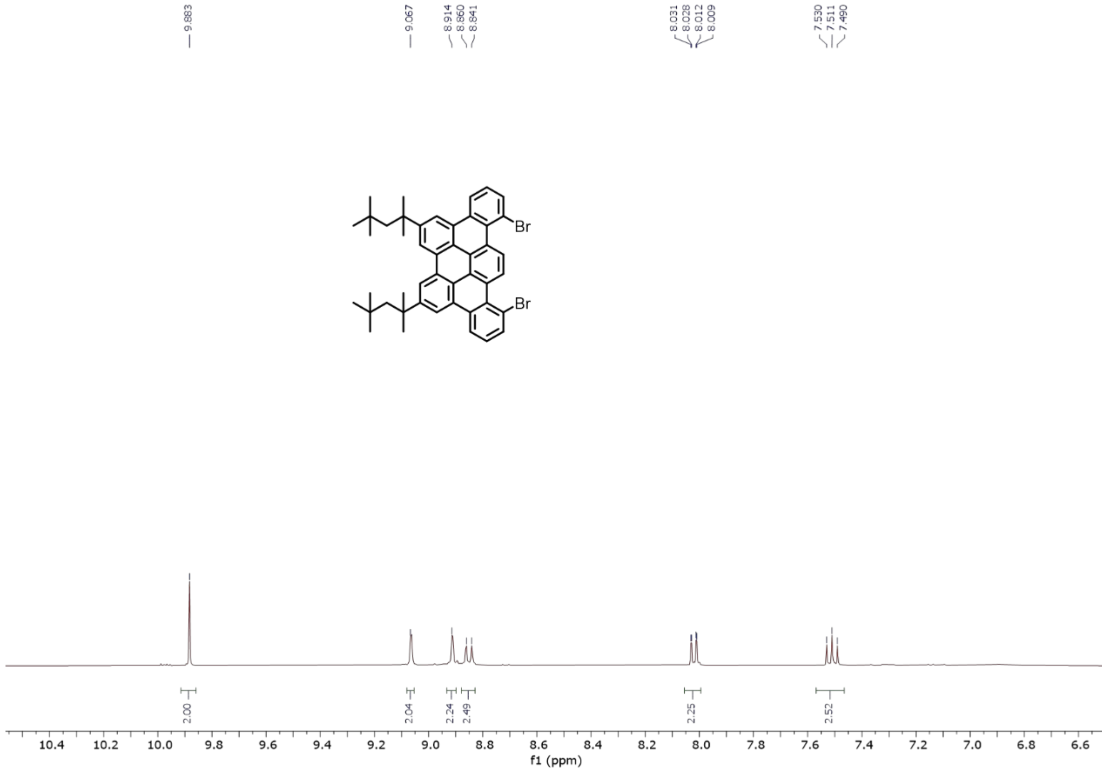

Figure S23. ^{1}H NMR spectrum and magnified spectrum at the aromatic region of compound **9b** in CD_2Cl_2 (400 MHz).

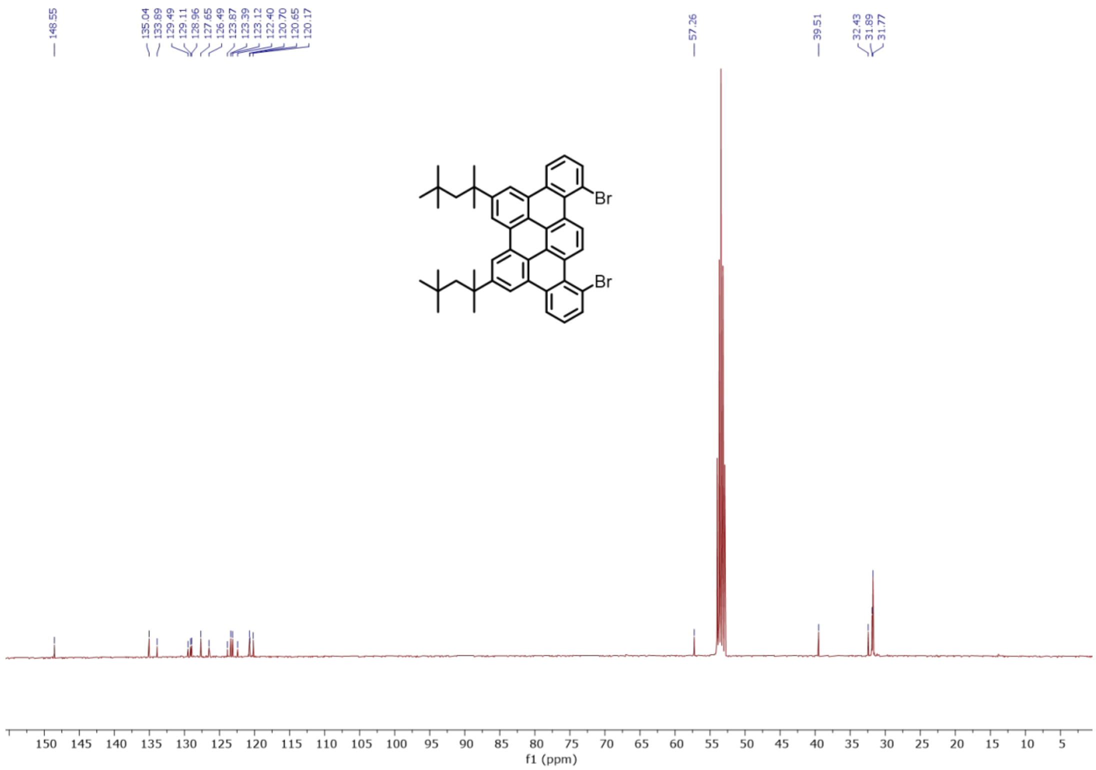

Figure S24. ^{13}C NMR spectrum of compound **9b** in CD_2Cl_2 (101 MHz).

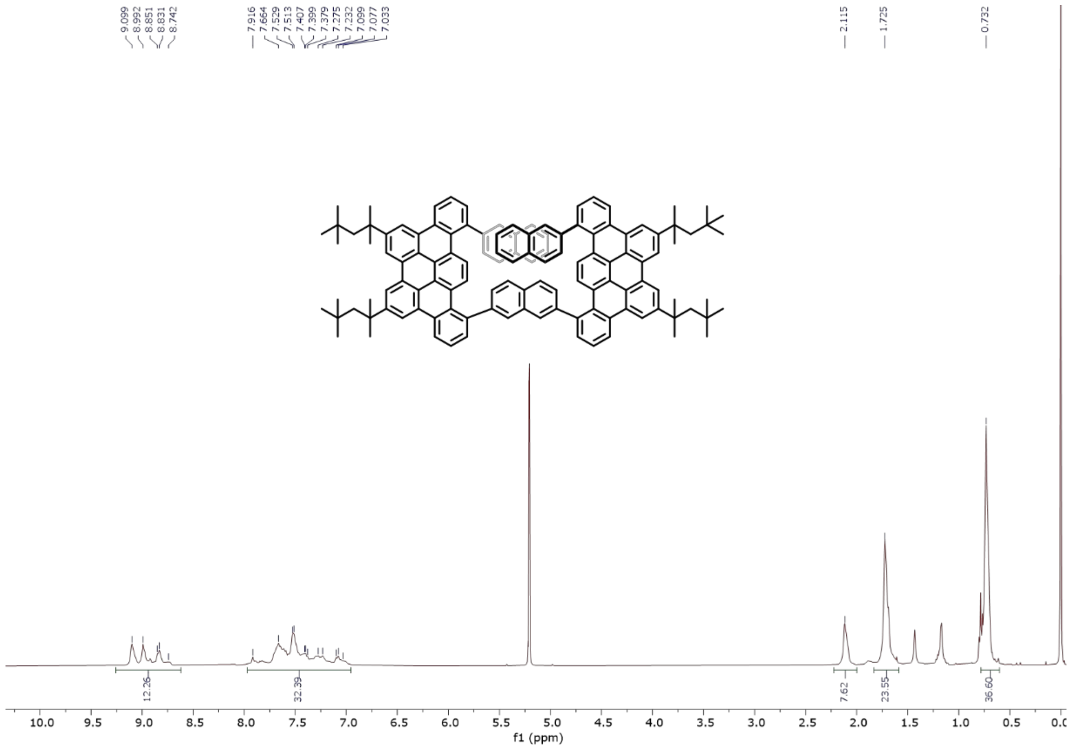

Figure S25. ^{1}H NMR spectrum of compound **1b** in CD_2Cl_2 (400 MHz).

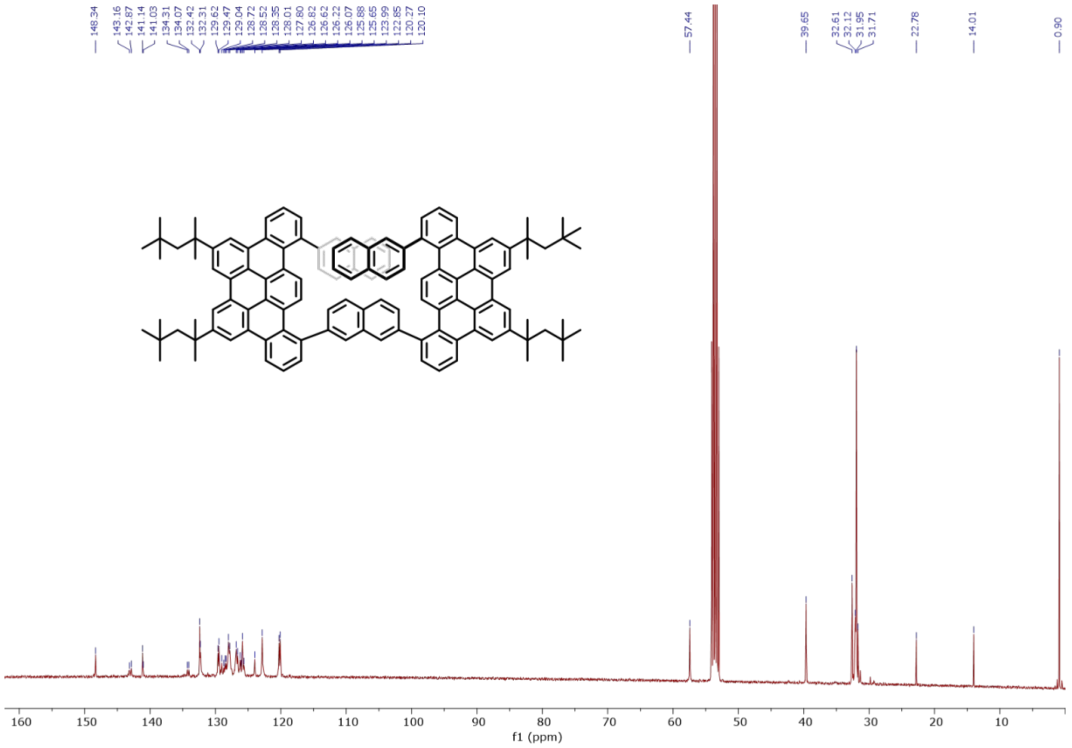

Figure S26. ^{13}C NMR spectrum of compound **1b** in CD_2Cl_2 (101 MHz).

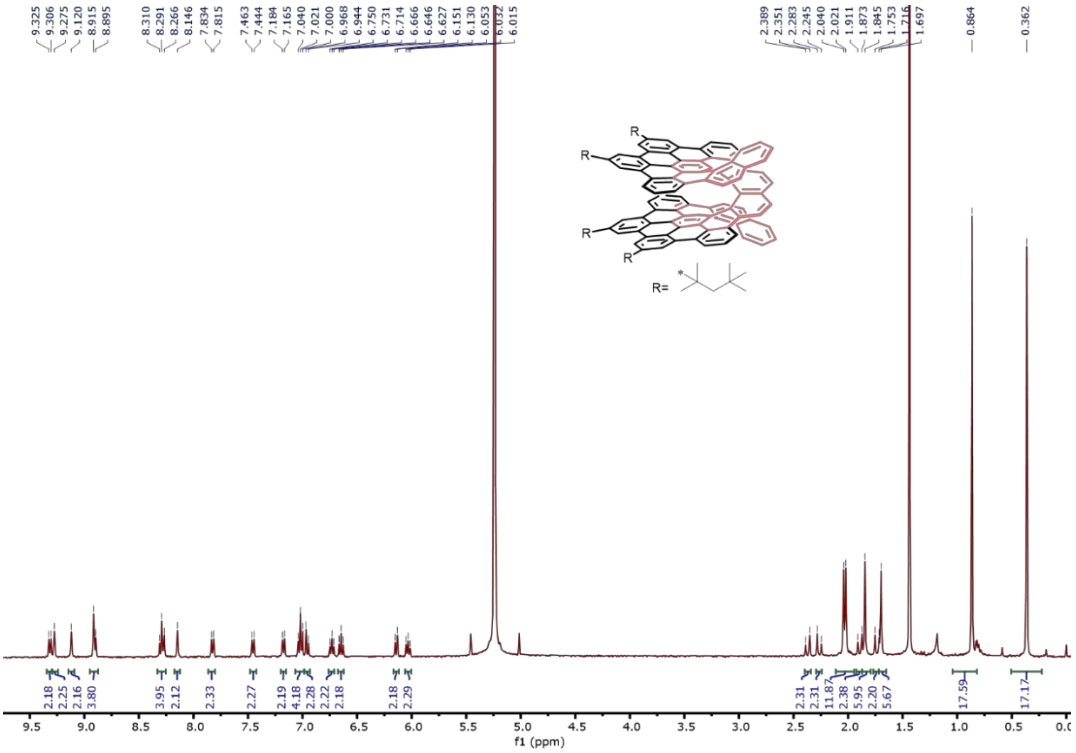

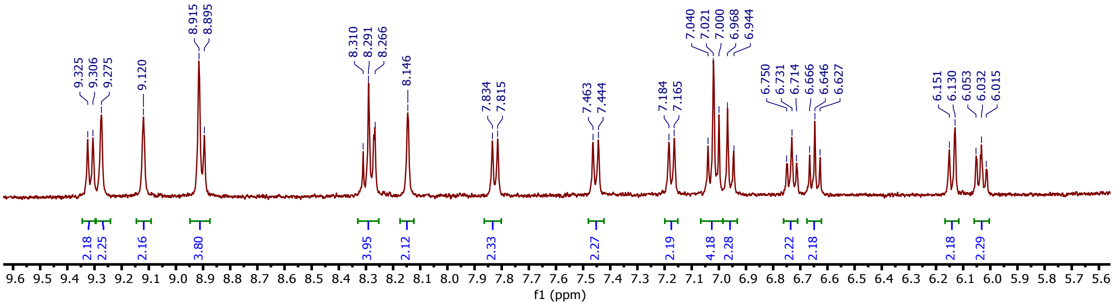

Figure S27. ^{1}H NMR spectrum and magnified spectrum at the aromatic region of E[12]H **2b** in CD_2Cl_2 (400 MHz).

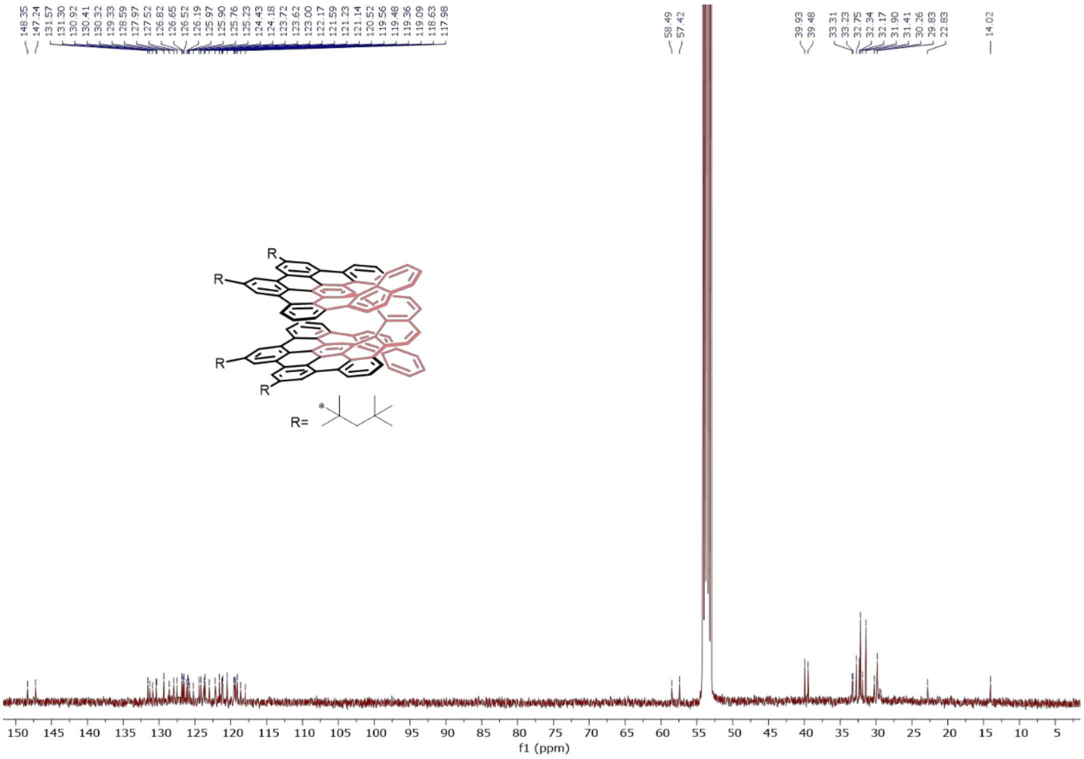

Figure S28. ^{13}C NMR spectrum of compound E[12]H **2b** in CD_2Cl_2 (101 MHz).

11. References

1. Di Giovannantonio, M. *et al.*, On-Surface Growth Dynamics of Graphene Nanoribbons: The Role of Halogen Functionalization. *ACS Nano* **2017**, *12*, 74-81.
2. Gu, Y. *et al.*, Cove-Edged Nanographenes with Localized Double Bonds. *Angew. Chem. Int. Ed.* **2020**, *59*, 8113-8117.
3. Yao, X. *et al.*, Synthesis of Nonplanar Graphene Nanoribbon with Fjord Edges. *J. Am. Chem. Soc.* **2021**, *143*, 5654– 5658.
4. Wang, J. *et al.*, Synthesis of a magnetic pi-extended carbon nanosolenoid with Riemann surfaces. *Nat. Commun.* **2022**, *13*, 1239.
5. Tommasini, M. *et al.*, Fingerprints of polycyclic aromatic hydrocarbons (PAHs) in infrared absorption spectroscopy. *Spectrochim. Acta, Part A* **2016**, *152*, 134-148.
6. Yao, X. *et al.*, N=8 Armchair Graphene Nanoribbons: Solution Synthesis and High Charge Carrier Mobility. *Angew. Chem. Int. Ed.* **2023***,* *62*, e202312610.
7. Hinaut, A., *et al.*, Electrospray deposition of organic molecules on bulk insulator surfaces. *Beilstein J. Nanotechnol.* **2015**, *6*, 1927-1934.
8. Shiraki, T. *et al.*, Defect Photoluminescence from Alkylated Boron Nitride Nanotubes. *Chem. Lett.* **2023**, *52*, 44-47.
9. Okada, S. *et al.*, Direct Microscopic Analysis of Individual C 60 Dimerization Events: Kinetics and Mechanisms. *J. Am. Chem. Soc.* **2017**, *139*, 18281-18287.
10. Shimizu, T. *et al.*, Real-Time Video Imaging of Mechanical Motions of a Single Molecular Shuttle with Sub-Millisecond Sub-Angstrom Precision. *Bull. Chem. Soc. Jpn.* **2020***,* 93, 1079-1085.
11. Hosokawa, F. *et al.*, Benchmark Test of Accelerated Multi-Slice Simulation by GPGPU. *Ultramicroscopy* **2015**, *158*, 56-64.
12. Gaussian 16, Revision C.01, M. J. Frisch, G. W. Trucks, H. B. Schlegel, G. E. Scuseria, M. A. Robb, J. R. Cheeseman, G. Scalmani, V. Barone, G. A. Petersson, H. Nakatsuji, X. Li, M. Caricato, A. V. Marenich, J. Bloino, B. G. Janesko, R. Gomperts, B. Mennucci, H. P. Hratchian, J. V. Ortiz, A. F. Izmaylov, J. L. Sonnenberg, Williams, F. Ding, F. Lipparini, F. Egidi, J. Goings, B. Peng, A. Petrone, T. Henderson, D. Ranasinghe, V. G. Zakrzewski, J. Gao, N. Rega, G. Zheng, W. Liang, M. Hada, M. Ehara, K. Toyota, R. Fukuda, J. Hasegawa, M. Ishida, T. Nakajima, Y. Honda, O. Kitao, H. Nakai, T. Vreven, K. Throssell, J. A. Montgomery Jr., J. E. Peralta, F. Ogliaro, M. J. Bearpark, J. J. Heyd, E. N. Brothers,

K. N. Kudin, V. N. Staroverov, T. A. Keith, R. Kobayashi, J. Normand, K. Raghavachari, A. P. Rendell, J. C. Burant, S. S. Iyengar, J. Tomasi, M. Cossi, J. M. Millam, M. Klene, C. Adamo, R. Cammi, J. W. Ochterski, R. L. Martin, K. Morokuma, O. Farkas, J. B. Foresman, D. J. Fox, Gaussian, Inc., Wallingford, CT, **2016**.

13. Becke, A. D., Density-functional thermochemistry. III. The role of exact exchange. *J. Chem. Phys*. **1993**, 98, 5648-5652.
14. Neese, F. *et al.,* The ORCA quantum chemistry program package. *J. Chem. Phys.* **2020**, *152*, 224108.
15. Lu, T. *et al.,* Multiwfn: A multifunctional wavefunction analyzer. *J. Comput. Chem.* **2012**, *33*, 580–592.
16. Humphrey, W. *et al.,* VMD: Visual molecular dynamics. *J. Mol. Graph. Model.* **1996**, *14*, 33–38.
17. Giannozzi, P. *et al.*, QUANTUM ESPRESSO toward the exascale. *J. Chem. Phys.* **2020**, *152***,** 154105.
18. Prandini, G. *et al.*, Precision and efficiency in solid-state pseudopotential calculations. *NPJ Comput. Mater.* **2018**, *4*, 72.
19. Klimes, J. *et al.*, Van der Waals density functionals applied to solids. *Phys. Rev. B* **2011**, *83*, 195131.
20. Contreras-García, J. *et al.*, NCIPLOT: A program for plotting noncovalent interaction regions. *J. Chem. Theory Comput.* 2011, *7*, 625–632.
21. Porsev, V. V. et al., Ab initio modeling of helically periodic nanostructures using CRYSTAL17: A general algorithm first applied to nanohelicenes. *Comput. Mater. Sci.*, **2022**, *203***,** 111063.
22. Smith, N. V., Classical generalization of the Drude formula for the optical conductivity. *Phys. Rev. B* **2001**, *64*, 155106.
23. Smith, N. V., Drude theory and the optical properties of liquid mercury. *Phys. Lett. A* **1968**, *26*, 126-127.
24. Hendry, E. *et al.*, Efficiency of Exciton and Charge Carrier Photogeneration in a Semiconducting Polymer. *Phys. Rev. Lett.* **2004**, *92*, 196601.
25. Wang, X. *et al.*, Cove-Edged Graphene Nanoribbons with Incorporation of Periodic Zigzag-Edge Segments. *J. Am. Chem. Soc.* **2022**, *144*, 228-235.
26. Liu, K. *et al.,* Cove-Edged Chiral Graphene Nanoribbons with Chirality-Dependent Bandgap and Carrier Mobility. *J. Am. Chem. Soc.* **2024**, *146*, 1026-1034.

27. Götz, A. *et al.,* Band structure modulation by methoxy-functionalization of graphene nanoribbons. *J. Mater. Chem. C* **2022**, 10, 4173-4181.
28. Yang, L. *et al.,* Solution Synthesis and Characterization of a Long and Curved Graphene Nanoribbon with Hybrid Cove-Armchair-Gulf Edge Structures. *Adv. Sci.* **2022**, *9*, e2200708.
29. Obermann, S. *et al.,* Curved graphene nanoribbons derived from tetrahydropyrene-based polyphenylenes via one-pot K-region oxidation and Scholl cyclization. *Chem. Sci.* **2023**, *14*, 8607-8614.
30. Yao, X. *et al.,* Synthesis of Nonplanar Graphene Nanoribbon with Fjord Edges. *J. Am. Chem. Soc.* **2021**, *143*, 5654-5658.
31. Unruh, M. T. *et al.,* Two Regioisomeric Ladder Polymers with a Fully Conjugated or Cross-Conjugated Polyacene-Type Skeleton. *Macromolecules* **2024**, *57*, 6390-6395.
